# The Function of the HGF/c-Met Axis in Hepatocellular Carcinoma

**DOI:** 10.3389/fcell.2020.00055

**Published:** 2020-02-07

**Authors:** Haiyu Wang, Benchen Rao, Jiamin Lou, Jianhao Li, Zhenguo Liu, Ang Li, Guangying Cui, Zhigang Ren, Zujiang Yu

**Affiliations:** ^1^Department of Infectious Diseases, The First Affiliated Hospital of Zhengzhou University, Zhengzhou, China; ^2^Gene Hospital of Henan Province, Precision Medicine Center, The First Affiliated Hospital of Zhengzhou University, Zhengzhou, China

**Keywords:** hepatocellular carcinoma, HGF/c-Met axis, function, molecule target therapy, sorafenib

## Abstract

Hepatocellular carcinoma (HCC) is one of the most common malignancies worldwide, leading to a large global cancer burden. Hepatocyte growth factor (HGF) and its high-affinity receptor, mesenchymal epithelial transition factor (c-Met), are closely related to the onset, progression, and metastasis of multiple tumors. The HGF/c-Met axis is involved in cell proliferation, movement, differentiation, invasion, angiogenesis, and apoptosis by activating multiple downstream signaling pathways. In this review, we focus on the function of the HGF/c-Met axis in HCC. The HGF/c-Met axis promotes the onset, proliferation, invasion, and metastasis of HCC. Moreover, it can serve as a biomarker for diagnosis and prognosis, as well as a therapeutic target for HCC. In addition, it is closely related to drug resistance during HCC treatment.

## Introduction

Hepatocellular carcinoma (HCC) is the sixth most common cancer and the fourth leading cause of cancer-related death in the world (Ferlay et al., 2019), and its burden is expected to increase in the next few years. The main cause of HCC is chronic liver disease caused by hepatitis virus B and/or C. However, other factors are also related to the occurrence of HCC, including alcohol-related liver disease, obesity, type 2 diabetes, and mellitus-related non-alcoholic fatty liver disease (Dyson et al., 2014; Williams et al., 2014). Only about 40% of patients with early-stage or localized HCC are suitable for potentially curative treatments (surgical resection, liver transplantation, and local radiofrequency ablation) and 20% are suitable for transcatheter arterial chemoembolization (TACE) if in an intermediate stage (Giordano and Columbano, 2014). Due to lack of early effective diagnosis, around 80% patients are diagnosed with advanced HCC. The prognosis of advanced HCC is poor, the median overall survival (OS) time is roughly 1–2 months (Ren et al., 2020) and only systemic therapy can improve survival time.

Systemic treatments with sorafenib or lenvatinib followed by regorafenib, nivolumab, or ramucirumab have been demonstrated to improve survival (Forner et al., 2018). Among them, sorafenib [against c- rapidly accelerated fibrosarcoma (c-RAF), b-RAF, vascular endothelial growth factor receptor (VEGFR), c-KIT, and platelet-derived growth factor receptor] (Wilhelm et al., 2006; Giordano and Columbano, 2014) was the first drug for first-line treatment of advanced HCC (Marisi et al., 2019; Musso and Beraza, 2019), until the appearance of lenvatinib [against VEGFR/rearranged during transfection proto-oncogene (RET)/fibroblast growth factor receptor (FGFR)] (Kudo et al., 2018). Second-line treatments have also been designed over the past few years, including regorafenib (antiangiogenesis) (Bruix et al., 2017), nivolumab [against the programed cell death-1 (PD1) immune checkpoint] (El-Khoueiry et al., 2017), and ramucirumab (against VEGFR2) (Zhu et al., 2019). For sorafenib used in first-line therapy, results of a randomized, placebo-controlled, phase III clinical trial investigating efficacy of sorafenib in 271 patients with HCC have been reported. The primary endpoint, median OS was 6.5 months for patients with sorafenib and 4.2 months for patients with placebo (Cheng et al., 2009). Another recent study demonstrated that OS was higher in patients treated with sorafenib (10.7 months) than patients who received the placebo (7.9 months) (Llovet et al., 2008). However, the survival benefit of sorafenib was disappointing in clinical trials, owing to its low response rate and short effective duration, caused by intrinsic and acquired resistance (Cheng et al., 2009; Llovet et al., 2015). Therefore, we need to explore the resistance mechanism of sorafenib and novel treatments against HCC, such as c-Met and downstream signaling pathway inhibitors (Hu et al., 2017).

**FIGURE 1:**
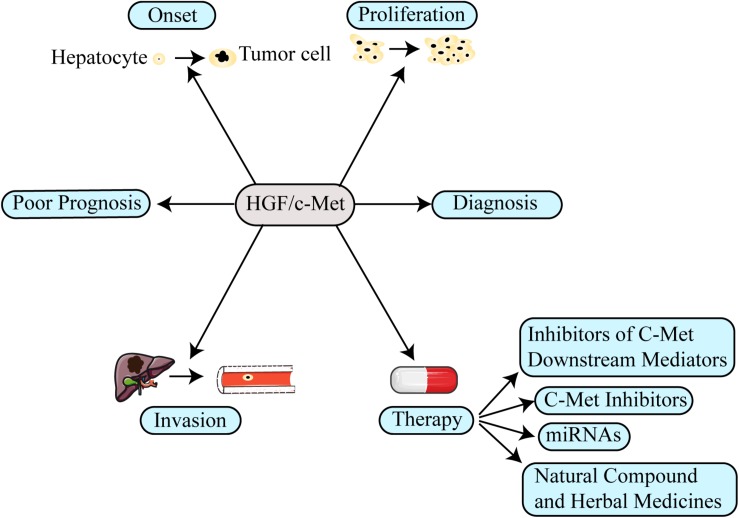
The function of the HGF/c-Met in hepatocellular carcinoma (HCC). The aberrant activation of HGF/c-Met axis involves several aspects in HCC, including promoting tumor onset, proliferation, invasion and even inducing drug resistance. Moreover, the HGF/c-Met axis could be used as prognosis and diagnosis biomarker for HCC. Therefore, targeting its mechanism in the formation of HCC, several new therapeutic strategies have been designed to treat patients with HCC.

Hepatocyte growth factor (HGF) and its high-affinity receptor, mesenchymal epithelial transition factor (c-Met), are closely related to the onset, progression and metastasis of multiple tumors. The HGF/c-Met axis has a pivotal role in normal liver growth, regeneration, and protection (Giordano and Columbano, 2014). Inappropriate Met activation promotes the onset, proliferation, invasion, and metastasis of HCC (Figure 1) via canonical or non-canonical pathways to regulate cell proliferation, movement, differentiation, invasion, angiogenesis, and anti-apoptosis. HCC patients with high expression of Met have lower survival time than patients with low or no expression of Met (Boccaccio and Comoglio, 2006). Moreover, the HGF/c-Met axis is regarded as a tumor aggressiveness and prognosis biomarker of patients with HCC (Garcia-Vilas and Medina, 2018). In addition, c-Met plays a critical role in drug resistance (Maroun and Rowlands, 2014). Therefore, targeting HGF/c-Met axis is one of the most promising therapies for HCC (You et al., 2011; Gao et al., 2012, 2017; Goyal et al., 2013; Graveel et al., 2013; Giordano and Columbano, 2014; Blagotinsek and Rozman, 2017).

## The Hgf/c-Met Axis

Hepatocyte growth factor was originally discovered in *in vitro* experiments as a hepatocyte mitogen (Nakamura et al., 1984) for primary hepatocytes that promoted the cell motility of epithelial cells (Stoker et al., 1987). Subsequently, several studies also revealed other effects, such as intensifying cell motility, angiogenesis, immune response, cell differentiation, and anti-apoptosis (Garcia-Vilas and Medina, 2018). Hepatocyte stromal cells or HCC tumor cells can express and release HGF into the tumor microenvironment (Matsumoto et al., 2008). HGF binds to its specific receptor, c-Met, which is located on the surface of hepatocytes, in a paracrine or autocrine manner. Moreover, the autocrine and paracrine activation of c-Met play an important role in the development and metastasis in HCC (Xie et al., 2001).

Originally, in a chemically transformed human osteosarcoma cell line, researchers found the c-Met proto-oncogene and identified it as a fusion gene (Cooper et al., 1984). It encodes the receptor for the ligand HGF. Several kinds of cells express c-Met, such as epithelial cells, neurons, hepatocytes, and hematopoietic cells (Fasolo et al., 2013). C-Met is a receptor tyrosine kinase (RTK) that is composed of a disulfide-linked heterodimeric complex. The complex is a transmembrane monomer that has five catalytic tyrosines in a cytoplasmic tail with four distinct hotspots (Basilico et al., 2014). One of five catalytic tyrosine regulates c-Met negatively (Y1003), while the others (Y1234, Y1235, Y1349, Y1356) (Bradley et al., 2017) regulate c-Met positively. Y1003 regulates Cbl-mediated Met lysosomal degradation. Activated Y1234 and Y1235 upregulate kinase activity and result in phosphorylation of the docking site residues Y1349 and Y1356, leading to the recruitment of adaptor proteins and signaling molecules.

Additionally, protein kinase-c activates S985 to degenerate c-Met. Hotspots are the domains of Met responsible for interaction with HGF. For four hotspots, the first hotspot is located on blades 2–3 of the semaphoring (SEMA) homology domain β-propeller, the known HGF β chain binging site. The second and third hotspot are known as the HGF α chain, localized on blade five of the SEMA domain and immunoglobulin-plexin-transcription factor (IPT) homology domains 2–3, respectively. The fourth hotspot is not previously correlated with the HGF binding site, which is across the plexinsemaphorin-integrin homology domain (PSI)-IPT 1 domains. C-Met, activated by the canonical pathway or the non-canonical pathway is involved in cell proliferation, motility, angiogenesis, invasion, and apoptosis.

### Canonical Mode of c-Met Activation Pattern

**FIGURE 2:**
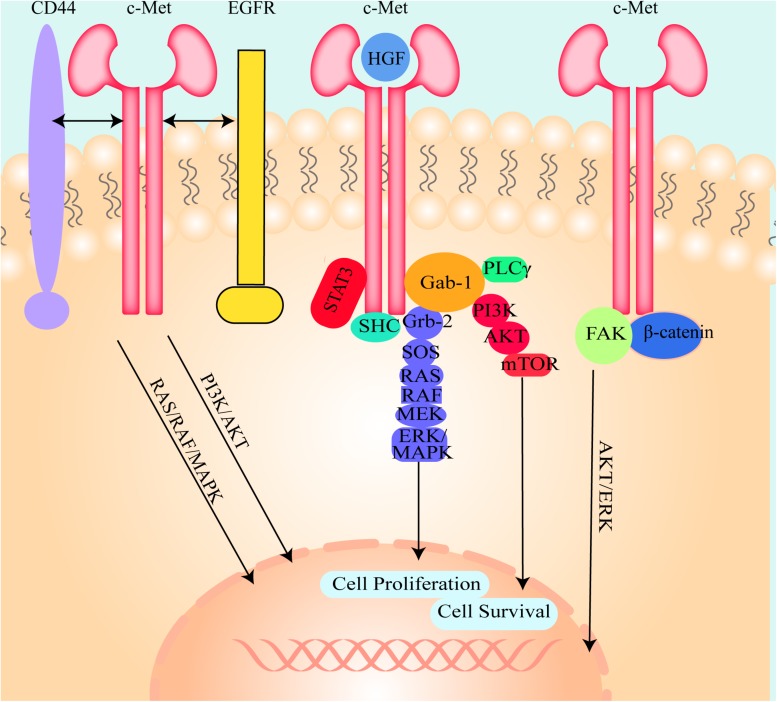
The illustration of the molecule mechanism of HGF/c-Met downstream signaling pathways and the crosstalk between c-Met and other cell signal transduction pathways. HGF binds to c-Met and induces c-Met homodimerization and autophosphorylation, then activates Gab-1, Grb-2, SHC, and STAT3. Grb2 activates SOS, SOS stimulates RAS and then RAS activates RAF, MEK, and ERK/MAPK. Activated ERK/MAPK can enter into the nucleus and modulate transcription factors to regulate cell behaviors. Activated Gab-1 stimulates AKT, PKB and mTOR to regulate transcription factors. C-Met could also interact with other cell signal transduction pathways such as CD44, EGFR, FAK, and β-catenin to regulate cell behaviors.

The canonical mode of c-Met activation involves the binding of HGF to c-Met, which induces homodimerization and autophosphorylation of the cytoplasmic domain of c-Met and then triggers downstream signaling pathways, including the mitogen-activated protein kinase (MAPK)/extracellular signal-related kinase (ERK) (Gonzalez et al., 2017), phosphatidylinositol 3 kinase (PI3K) (Pascale et al., 2016), p-38 (Lee et al., 2003), and the Akt/protein kinase B (PKB) (Zhang et al., 2018c) pathways (Figure 2). Many studies have shown that these signaling pathways are shared with other RTKs (Corso and Giordano, 2013). Adaptor proteins involved in these signaling pathways include, growth factor bound protein2 (Grb-2), Grb-2 associated binding protein 1 (Gab1), PI3K, signal transducer and activator of transcription 3 (STAT3), SH2-containing inositol 5-phosphatase 1 (SHIP1), phospholipase Cγ (PLCγ), SH2 domain-containing tyrosine phosphatase 2 (SHP2) (Liu et al., 2018), and Src homology and collagen homology (Shc) (Saucier et al., 2004). These proteins induce hepatocarcinogenesis and contain Src-homology 2 (SH2) domains or phosphotyrosine-binding domains and Src homology 3 (SH3) domains (Bradley et al., 2017; Garcia-Vilas and Medina, 2018), and are directly or indirectly bound to c-Met.

### Non-canonical Mode of c-Met Activation Pattern

C-Met can also be inappropriately activated by other pathways. Deregulated Met activation can induce several types of tumors in humans. (I) Des-γ-carboxy prothrombin (DCP) is secreted from HCC cells and activates c-Met because it contains two structural regions that are similar to HGF (Suzuki et al., 2005; Zhang Y.S. et al., 2014). Due to this similarity, DCP can bind to and activate c-Met. Moreover, DCP is used as a tumor screening and diagnostic biomarker owing to its sensitivity and specificity. (II) C-Met is modulated through crosstalk with different membrane receptors, including epidermal growth factor receptor (EGFR), human epidermal growth factor receptor (HER), Integrin, β- catenin, cluster of differentiation-44 (CD44), intercellular adhesion molecule-1 (ICAM-1), Plexin B1, VEGF-A, insulin receptor (INSR), FAS, Mucin 1 (MUC1), neuropilin (Nrp)-1 and -2, and focal adhesion kinase (FAK) (Figure 2; Jo et al., 2015; Garcia-Vilas and Medina, 2018). Although this crosstalk is not necessary for cell survival, it is able to better integrate the signals presented in the extracellular environment. Even if the crosstalk is redundant in physiological conditions, these interacting receptors may collaborate with each other in promoting tumorigenesis and/or metastasis and even cause resistance to target drugs in pathologic conditions. (III) C-Met overexpression drives the receptor activation and is induced by a few factors, including hypoxia (Pennacchietti et al., 2003; Ghiso and Giordano, 2013), inactivation of tumor suppressor genes, activation of upstream oncogenes and loss of miRNAs (Corso and Giordano, 2013). (IV) C-Met mutations can activate the receptor, thereby altering substrate specificity or catalytic activity. The identification of germline activating mutations in hereditary papillary renal carcinomas is unequivocal evidence correlating Met with cancer (Schmidt et al., 1999). (V) C-Met can also be activated by amplification. It has been shown that non-canonical pathways are associated with tumor progression, metastasis (Garcia-Vilas and Medina, 2018) and drug resistance (Migliore and Giordano, 2008; Scagliotti et al., 2013) in *in vivo* experiments. (VI) Autocrine Met-induced-activation is due to ectopic Met expression in cells yielding HGF, especially in acute myeloid leukemia (Kentsis et al., 2012). (VII) miRNAs directly degrade messenger RNA or repress translation to regulate gene expression (Friedman et al., 2009). Deregulated miRNA expression in HCC tissues has been detected. (VIII) Long non-coding RNAs (lncRNAs) could modulate c-Met expression by interacting with miRNAs (Zhang et al., 2019). (IX) Slug can mediate activation of c-Met in a ligand-independent manner because of increased levels of fibronectin and induced integrin α V function (Chang et al., 2019).

The HGF/c-Met axis has an important role in cellular behaviors, such as cell proliferation, migration, survival, morphogenesis and the epithelial–mesenchymal transition (EMT) (Taher et al., 2002; Bouattour et al., 2018). Moreover, it also is essential for liver formation, growth, regeneration, protection, and angiogenesis during embryonic development and in adulthood after injury (Borowiak et al., 2004). In HGF and c-Met knockout mice, mice are embryonic lethal due in part to impaired liver formation (Schmidt et al., 1995). After partial hepatectomy, HGF expression levels increased quickly in rodents (Nakamura et al., 1984), and mice that conditionally inactivate c-Met in mature hepatocytes show insufficient liver regeneration (Borowiak et al., 2004). However, the aberrant activation of HGF/c-Met signaling pathways, such as c-Met over-expression, amplification, binding to other ligands or abnormally high HGF levels, leads the initiation and progression of tumors, such as non-small cell lung cancer, HCC, colon cancer, renal caner, and breast cancer (Goyal et al., 2013; Li J. et al., 2019). Furthermore, both the canonical and non-canonical signaling pathways require the dimerization and autophosphorylation of c-Met. Therefore, c-Met is the key factor in the HGF/c-Met signaling pathways. C-Met and its downstream signal mediators are promising targets in treating patients with advanced HCC.

## The Hgf/c-Met Axis in Hcc

Numerous *in vivo* and *in vitro* studies have demonstrated that HGF/c-Met play a critical role in the development of various human cancers (renal, lung, liver, breast, colon, thyroid, ovarian, and pancreas). HGF/c-Met signaling pathways are uncontrolled in human cancer via overexpression of HGF or c-Met, gene amplification, mutational activation of c-Met, down-regulation of Met-targeted miRNA, binding to other ligands, autocrine signaling, or abnormally high HGF levels. Deregulated activation of c-Met contributes to a few aspects of tumor progression, such as inducing neoplastic cells to disaggregate from the tumor mass, eroding basement membranes, infiltrating stromal matrices, and finally colonizing new tissues to form metastases (Corso and Giordano, 2013). Here we mainly discuss the HGF/c-Met axis in HCC.

### Onset

Chronic liver diseases such as cirrhosis and hepatitis B or C are triggers of HCC (Janevska et al., 2015). There is a complicated interplay between HCC, chronic liver diseases and c-Met. Liver diseases reduce hepatocytes and increase the need for hepatocyte proliferation, thereby promoting up-regulation of c-Met and/or HGF. The increasing c-Met levels induce hepatocyte proliferation, regeneration, and survival during liver repair and delay the development of liver diseases by repressing chronic inflammation and the progression of fibrosis. Although it is potentially beneficial for liver diseases, increased c-Met activity can initiate, drive or promote the progression of HCC (Bouattour et al., 2018). Reversely, a knockout of the c-Met increased chemically-mediated HCC initiation but did not affect phenobarbital-induced HCC promotion (Marx-Stoelting et al., 2009). Moreover, the intact and normal HGF/c-Met signaling is elementary for sustaining normal redox homeostasis and could suppress tumor in the *N*-nitrosodiethylamine-induced HCC (Takami et al., 2007). Additionally, c-Met may induce VEGF-A expression, which can enhance tumor angiogenesis (Li et al., 2018; Zhang et al., 2018b).

As mentioned above, c-Met is aberrantly activated by gene amplification, overexpression, mutation, binding to other ligands, autocrine signaling or abnormally high HGF levels in cancer. However, according to the study by Takeo et al. (2001), Met amplification was at a very low frequency in HCC (one-twentieth). In the study by Kondo, the amplification frequency is 159th (Kondo et al., 2013). Concerning activating Met kinase domain mutations, Park and Di Renzo and Lee and Aebersold (Park et al., 1999; Di Renzo et al., 2000; Lee et al., 2000; Aebersold et al., 2003) observed three missense mutations in childhood HCC (K1262R, M12681, T11911, respectively) (Park et al., 1999). Mutations in the Casitas B-cell lymphoma (Cbl)-binding domain are demonstrated to be oncogenic because binding of Cbl to Y1003 causes Met ubiquitination that is vital to maintenance of physiological Met activation and prevention of continued activation of Met (Abella et al., 2005; Peschard and Park, 2007). Under normal conditions, activated Met is rapidly removed from the cell surface by ubiquitination, and then targets the lysosomal degradation chamber. More and more evidence shows that the ubiquitination of RTK is the key to its lysosomal degradation and recruitment of the ubiquitin protein ligase Cbl family is required for ligand-induced degradation of many RTKs. Moreover, the phosphorylation of Y1003 provides a direct docking site for the SH2-like crystal structure of Cbl (TKB) domain of Cbl ubiquitin ligase and is required for ligand-dependent ubiquitination and Met receptor degradation. Therefore, Y1003 mutations in the cbl-binding region can lead to the continued activation of Met and its downstream signaling pathways, and even induce cancer. Furthermore, mutations in the juxtamembrane domain led to tumorigenesis in an *in vitro* trial (Graveel et al., 2013). Levels of c-Met were higher (or showed overexpression) in 20–48% of HCC samples than levels in peritumoral liver tissue (Tavian et al., 2000). Over-expression of c-Met occurs more often than mutation and amplification. While not all HCC are related to HGF or c-Met overexpression (Zhang et al., 2005), HCC patients with c-Met overexpression have poor prognosis. The expression of HGF is decreased in HCC, but is increased in peritumoral liver tissue (Garcia-Vilas and Medina, 2018). The increased secretion of HGF in the peritumoral liver tissue may be due to the increased release of HGF from hepatic stellate cells to the peritumoral liver tissue. While the decreased secretion of HGF in HCC tissue may be due to the HGF from HCC cells directly bounding to c-Met through autocrine pathway.

Additionally, microRNAs (miRNAs) and suppressor of cytokine signaling 1 (SOCS1) (Gui et al., 2011) can control hepatocarcinogenesis by regulating HGF/c-Met. Gui et al. (2015, 2017) have demonstrated that SOCS1 overexpression can inhibit HCC cells proliferation and migration by attenuating HGF-induced phosphorylation of c-Met, Gab1, and ERK1/2. MiR-181a can inhibit hepatocarcinogenesis through repressing activation of c-Met (Korhan et al., 2014). Therefore, up-regulation of oncogenic miRNA induces HCC progression.

In addition to the above, cooperation of the HGF/c-Met pathway with MUCI (Bozkaya et al., 2012) or β-catenin (Tao et al., 2016) can induce hepatocarcinogenesis. Qiao et al. (2019) demonstrated that loss of axis inhibition protein (Axin1) cooperated with c-Met to cause HCC in mice. Similarly, loss of β-Catenin also exacerbated hepatocarcinogenesis driven by Met and oncogenic β-catenin (Liang et al., 2018).

A study carried out by Kaposi-Novak has found that a Met-regulated expression signature correlated vascular invasion rate and decreased mean survival time and microvessel density in a subset of human HCC and liver metastases (Kaposi-Novak et al., 2006). Wang et al. (2001) did a trial by using human Met transgenic mice to understand how ligand-independent activation of RTKs affects tumorigenesis. It was found that transgenic mice developed HCC, which subsided when the transgene was inhibited, which showed that Met over-expression induced tumorigenesis without HGF. The HGF/c-Met axis can also induce onset of HCC by promoting angiogenesis (Giordano and Columbano, 2014). In summary, although there are many ways to activate the c-met signaling pathway to induce the occurrence of HCC, c-met expression and activation are indispensable. Therefore, c-met is a therapeutic target that is worthy of research, and there are still many mechanisms of how HGF/c-Met signaling mediates tumorigenesis in HCC that we need to explore.

### Proliferation

Besides onset, the HGF/c-Met axis is also involved in proliferation of HCC. In 2005, to investigate the effects of c-Met expression on HCC cell growth Zhang et al. (2005) used an adenovirus-delivered small interfering RNA (siRNA) method to observe the knockdown of c-Met on tumorigenic growth of HCC in *in vitro* and *in vivo* trials In the *in vitro* trial, compared with adenovirus alcohol dehydrogenase (AdH1)-null or mock-infected cells, proliferation of MHCC97-L cells, which had high c-Met expression, were inhibited by adenovirus AdH1-siRNA, and c-Met expression also decreased. The MHCC97-L cells were arrested at G1-G0 phase. In the *in vivo* study, the proliferative indices of adenovirus AdH1-siRNA/Met-injected mouse tumors were lower (23.4%) than the adenovirus AdH1-null injected tumors (69.8%) and mock-injected tumors (72.8%). C-Met expression was obviously reduced by adenovirus AdH1-siRNA/Met injection. In addition, some studies have found lncRNAs can promote HCC cells proliferation, migration, and even invasion. According to the study of Zhang, lncRNA FLVCR1-AS1 sponges miR-513c and increases c-Met expression in HCC cells, which induces HCC progression (Zhang et al., 2018a). In another study, Zhang et al. (2019) has demonstrated that lncRNA HULC promotes HCC progression by inhibiting miR-2052 expression and activating c-Met signaling pathway. However, another study found that HGF plays a crucial role in HCC proliferation induced by cancer-associated fibroblasts from HCC (H-CAFs) in *in vitro* and *in vivo* trials (Jia et al., 2013). Tumor volume growth was consistent with HGF production. Furthermore, the effect of H-CAF conditioned medium on proliferation of HCC cells was significantly reduced by anti-HGF. Therefore, according to these studies, HGF/c-Met can induce proliferation of hepatocellular cells.

### Invasion and Metastasis

The high lethality of HCC results from primary tumors invading and migrating to other tissues. This process begins with tumors invading blood vessels and subsequently migrating into intrahepatic and extrahepatic tissues. Tumor metastasis is a complicated multistep process and invasion is a major element of this process, which includes damage of basement membranes and proteolysis of the extracellular matrix (ECM) (Liotta and Kohn, 2001). Several studies have reported that overexpression levels of HGF or c-Met in HCC correlate with incidence of invasion and metastasis and suggest that HGF/c-Met signaling had a crucial role in the invasion and metastasis of HCC cells (Ueki et al., 1997; Junbo et al., 1999; Wang et al., 2007). HGF/c-Met signaling pathways are involved in HCC cell invasion via HGF-induced c-Met phosphorylation, AKT phosphorylation, nuclear factor-κB (NF-κB) activation, and matrix metalloproteinase-9 (MMP-9) expression (Wang et al., 2007).

To investigate the invasion and metastasis effect of HGF/c-Met signaling in HCC, Liu and colleagues conducted a study using two different HGF-treated HCC cell lines, Hep3B and HepG2. The Hep3B HCC cell line was p53 deficient and overexpressed c-Met after treatment with HGF. Loss of p53 expression reinforced HGF/c-Met signaling, which promoted invasion and metastasis by upregulating Snail expression (Liu et al., 2016). Xie et al. (2010) found that overexpression of c-Met induces cell invasion. Other studies have also reported that peritumoral stromal neutrophils and mesenchymal cells secrete high levels of HGF, which drove high rates of proliferation, invasion and metastasis in HCC by promoting the EMT (Ding et al., 2010; He et al., 2016). Moreover, phenotypic analysis validated that mixed-lineage leukemia (MLL), an epigenetic regulator, interacts with HGF/c-Met signaling to induce invasion and metastatic growth of HCC cell lines (Marquardt and Thorgeirsson, 2013; Takeda et al., 2013).

The liver is an organ filled with blood vessels that rely on angiogenesis for cellular regeneration (Whittaker et al., 2010). Likewise, angiogenesis plays a critical role in tumor growth, invasion and metastasis (Semela and Dufour, 2004). The angiogenic balance between proangiogenic and antiangiogenic factors maintains normal angiogenesis (Semela and Dufour, 2004). However, the balance in HCC is disordered due to excessive angiogenic factors that are secreted by tumor cells, endothelial cells and pericytes. Many angiogenic factors, such as VEGF-A, HGF, transforming growth factor (TGF) and epidermal growth factor (EGF) (Folkman, 2003), demonstrated elevated expression levels in HCC tumors (Mas et al., 2007), Moreover, these factors induce angiogenesis through a number of mechanisms, one of them is via the HGF/c-Met signaling pathway. Several studies have reported that the HGF/c-Met axis induces angiogenesis and cell growth through interaction with the VEGF and VEGFR pathway and decreasing expression of thrombospondin-1 (Zhang et al., 2003; Abounader and Laterra, 2005).

## Diagnosis and Prognosis

Although new treatments have been used in HCC patients and provided possible cures, the long-term survival rate is still poor due to late diagnosis and high recurrence. Therefore, sensitive and specific diagnostic or prognostic biomarkers are urgently needed. Although the HGF/c-Met axis is an emerging study target, the possibility of its use in diagnosis and prognosis has been studied in addition to its mechanism in HCC.

In the study by Yamagamim et al. (2002), HCC patients had significantly increased serum levels of HGF than patients with chronic viral hepatitis C and cirrhosis. Thus, the serum HGF concentration may be helpful as a tumor biomarker for HCC. Likewise, Karabulut et al. (2014) and Zhuang et al. (2017) also identified serum HGF level as a potential diagnostic. However, Unic et al. (2018) demonstrated that the individual diagnostic performance of HGF was inadequate. Although the concentration of HGF is obviously higher in patients with alcoholic liver cirrhosis than in healthy humans, there is no significant difference in HGF serum level between cirrhosis patients with HCC and cirrhosis patients without HCC. It may be due to the reduction of hepatocytes in cirrhosis patient, which promotes the secretion of HGF and then activates c-Met to increase hepatocytes proliferation. Moreover, the diagnosis sensitivity of HGF was very high (90.62%) but the specificity was very low (25.81%). In conclusion, although the independent use of HGF for diagnosis is controversial, HGF is useful when combined with other diagnostic markers.

Some studies also suggested that the HGF/c-Met axis has prognostic value for patients with HCC (Zhuang et al., 2017; Garcia-Vilas and Medina, 2018). Overexpression of c-Met correlated with decreased 5-year survival in patients with HCC. In addition, the Met-driven expression signature defines a subset of HCC which has poor prognosis and an aggressive phenotype (Kaposi-Novak et al., 2006). Vejchapipat et al. (2004) has found that inoperable patients with HCC had higher levels of serum HGF than healthy humans due to the impaired clearance of HGF. Serum HGF concentration was negatively correlated with long-term survival time in HCC patients. Additionally, a serum HGF level of 1.0 ng/mL or more indicated a serious prognosis in patients with HCC. Nevertheless, another study (Ke et al., 2009) showed that c-Met was not an independent prognostic factor of HCC for OS and cumulative recurrence, but the combination of c-Met/CD151 was. Meanwhile, an other study (Gong et al., 2018) also suggested that the prognostic value of c-Met is contradictory. By univariate analysis, c-Met overexpression was significantly correlated with clinicopathological factors, but not with multivariate analysis. Furthermore, c-Met overexpression was not identified to be obviously correlated with OS rates in this study. The reason for the difference in these studies may be the small number of surveyed people or the different technique and scoring system. Thus, the role of HGF and c-Met as prognostic factors for HCC needs to be explored further in the future. Though, the combination of HGF and c-Met with other biomarkers may be useful in predicting the prognosis of HCC.

## Target Therapies

As mentioned above, there are five therapies that can prolong the expected lifespan of patients with HCC including, surgical resection, liver transplantation, local ablation, TACE and sorafenib. Only 40% percent of patients with early stage HCC are eligible for potentially curative treatments (surgical resection, transplantation, local ablation), which prolong median survival times over 60 months (Llovet et al., 2015). For patients with intermediate-stage HCC, TACE can improve estimated median survival by 26 months (Kudo et al., 2014). However, a large proportion of HCC patients are diagnosed at advanced stage and only systemic treatment with sorafenib can extend OS from 6 to 11 months (Llovet et al., 2008). Thus, sorafenib is regarded as the first-line treatment in advanced HCC patients with a manageable adverse event (Llovet et al., 2008). Nevertheless, in the past few years, seven randomized phase III clinical trials, which tested other first-line and second-line treatments, in intermediate-stage or advanced-stage HCC patients have not found any obvious OS benefits (Llovet et al., 2015). Moreover, the intrinsic or acquired resistance of sorafenib is the major obstacle in treatment. Thus, based on the understanding of the role of the HGF/c-Met signaling pathway and the uniqueness of c-Met in HCC, more and more therapeutic strategies target c-Met and the interaction between c-Met and downstream signaling mediators instead of the interaction between HGF and c-Met because of the varying activation of c-Met.

### c-Met Inhibitors

So far, there are six Met inhibitors developed and they have been tested in 10 HCC clinical trials (Bouattour et al., 2018). Small molecular kinase inhibitors can block phosphorylation of the catalytic domain in the receptor by competitive or non-competitive antagonism of the ATP binding site, thereby preventing the recruitment of signal transducers and mediators, and thus impeding the transmission of downstream signals (Munshi et al., 2010; Gao et al., 2012). Anti-c-Met agents can be categorized into three types: selective c-Met tyrosine kinase inhibitors (TKIs), multi-targeted TKIs including against c-Met, and monoclonal antibodies against HGF or c-Met (Goyal et al., 2013). A lot of preclinical studies have demonstrated the feasibility of HGF/c-Met as targets for the treatment of patients with HCC. For instance, AMG 337, a potential and highly selective small molecule Met kinase inhibitor, significantly decreases tumor growth of Met-high-expression and Met-amplified HCC cell lines in *in vitro* and *in vivo* trials (Du et al., 2016). Additionally, Indo5, selectively abrogating HGF-induced c-Met pathway activation and brain-derived neurotrophic factor (BDNF)/nerve growth factor (NDF)-induced Trks signaling activation, significantly inhibits HCC tumor growth in xenograft mice (Luo et al., 2019). Moreover, PHA665752 supressed cell proliferation, and induced apoptosis in MHCC97-L and MHCC97-H cells which overexpress c-Met through blocking phosphorylation of c-Met and downstream PI3K/Akt and MAPK/Erk pathways (You et al., 2011). PHA665752 also repressed MHCC97-L and MHCC97-H tumor growth in xenograft models.

Also, several clinical trials are (Garcia-Vilas and Medina, 2018) being carried out in HCC patients using c-Met inhibitors (Table 1), including cabozanitinib, foretinib, cobazitinib, gefitinib, crizotinib, MSC2156119, AZD4547, MK2461, and INC280 (Schiffer et al., 2005; Goyal et al., 2013; Bladt et al., 2014; Sun et al., 2017; Garcia-Vilas and Medina, 2018). Among them, cabozanitinib is undergoing randomized phase III clinical trials. In the latest clinical trial, OS and progression-free survival (PFS) in months was calculated for Phase 2 of Tepotinib 500 mg (5.55 and 3.22 months, respectively) (NCT02115373). In a randomized phase II study of axitinib, axitinib combined with best supportive care (BSC) did not improve OS versus placebo combined with BSC (Kang et al., 2015) (NCT01210495). Nevertheless, axitinib combined with BSC led to a significant prolongation of PFS and time to tumor progression (TTP) and an increase of clinical benefit rate (CBR), and the toxicity of patients with advanced HCC is acceptable.

**TABLE 1:** Drugs targeting HGF/c-Met in hepatocellular carcinoma patients.

Drug	Target	Phase	Activity	Type
Tivantinib	c-Met, tubulin	III	Failed	Non-selective TKI
Crizotinib	Met, ALK, ROS1	Ib	Anti-angiogenesis	Non-selective TKI
Cabozantinib	c-Met, KDR, RET, KIT, TIE-2, FLT-3	III	Anti-tumor	Non-selective TKI
Foretinib	Met, VEGFR2, KIT, FLT-3, PDGFR β, TIE-2	II	Anti-tumor	Non-selective TKI
Cobazitinib	c-Met, VEGFR-2, RET	II	Anti-tumor	Non-selective TKI
MSC2156119J	c-Met	Ib/II	Anti-tumor, Anti-metastasis	Selective TKI
Gefitinib	EGFR, c-Met, HGF	II	Anti-tumor	Non-selective TKI
MK2461	c-Met, Ftl-1	I	Anti-tumor	Non-selective TKI
Capmatinib	c-Met	II	Anti-tumor	Selective TKI
Tepotinib	c-Met	II	Anti-tumor	Selective TKI
Golvatinib	c-Met, KDR	II	Anti-tumor	Non-selective TKI

However, there is currently no effective method for treating HCC based on traditional monotherapy with TKIs. In a previous phase II randomized controlled clinical study, Tivantinib, a highly selective c-Met inhibitor, improved median time to progression and OS time in patients with Met-high advanced HCC compared with placebo (Santoro et al., 2013). However, in a randomized phase III double-blind clinical trial, tivantinib did not improve OS time in patients with Met-high advanced HCC treated with sorafenib compared with placebo (Rimassa et al., 2018) (NCT01755767). Despite the failure of tivantinib in a phase III clinical trial to achieve its primary endpoint, we could not deny the role of c-Met inhibitors in the treatment of liver cancer. The reason for this failure, in my opinion, may be due to tivantinib being a non-selective c-Met inhibitor rather a selective c-Met inhibitor. The cytotoxicity against many HCC cell lines of tivantinib was unrelated to c-Met expression but related to inhibiting microtubule assembly (Aoyama et al., 2014) and Glycogen Synthase Kinase-3 alpha (GSK3a) and beta (GSK3b) (Remsing Rix et al., 2014) in other studies. Inhibition of non-c-Met targets may enhance the antitumor activity of non-selective c-Met inhibitors, but is also correlated with increased toxicity and limits the dose so that inhibitors cannot effectively suppress c-Met (Bouattour et al., 2018). Furthermore, the increased toxicity and limitation of potential benefit may outweigh the enhanced antitumor activity because of inhibition of multiple targets. Moreover, we cannot attribute the anti-tumor effect of non-selective c-Met inhibitors to the inhibition of c-Met. Thus, selective c-Met inhibitors may be a better choice for treatment in patients and more studies are needed to identify the reason for phase II clinical trial failure and the feasibility of using c-Met targeting therapies.

### MicroRNAs

Fortunately, using potential miRNAs for suppressing aberrant c-Met signal is an emerging and promising therapy strategy that bypasses traditional approaches (Karagonlar et al., 2015) (Table 2). miRNAs are small non-coding RNAs and regulate gene expression by resolving mRNA or suppressing translation (Karagonlar et al., 2015). Previous studies have found that miRNA expression in cancer tissue is different from normal tissues (Murakami et al., 2006; Volinia et al., 2006; Ladeiro et al., 2008). miRNAs can not only inhibit tumor growth, proliferation, invasion, and metastasis but also induce many kinds of tumors. Herein, we mainly focus on the role of miRNAs in HCC. For example, miR-101 suppresses the proliferation and migration of HCC cells and tumors through targeting HGF/c-Met, Girdin, SOX9 and TGF-β in *in vitro* and *in vivo* trials (Cao et al., 2016; Yang et al., 2016; Yan et al., 2018; Liu et al., 2019). miRNA-206 targets c-Met and cyclin-dependent kinase 6 (Cdk6) to suppress development of HCC in mice (Wu et al., 2017). Also, miR-26a inhibits tumor growth, metastasis and angiogenesis of HCC via targeting HGF-induced c-Met signaling pathways and FBXO11, ST3GAL5 signaling pathways (Yang et al., 2014; Cai et al., 2017; Ma et al., 2018). MiR-93 induces HCC cell proliferation and invasion by activating c-Met/PI3K/Akt signaling pathways and targeting PDCD4 and TIMP2 (Ohta et al., 2015; Ji et al., 2017; Xue et al., 2018). Thus, we can design miRNA mimics to overexpress miRNAs that down-regulate c-Met signaling pathways, and design miRNAs antagonists to inhibit miRNAs that up-regulate c-Met signaling pathways (Karagonlar et al., 2015). Moreover, one of the major advantages of miRNA therapy is simultaneously targeting multiple effectors in several signaling pathways involved in tumorigenesis.

**TABLE 2:** miRNAs that target HGF/c-Met signaling in hepatocellular carcinoma.

miRNAs	Functions	Mechanisms	References
miR-34	Represses cell invasion, proliferation, migration promotes apoptosis	Upregulation of p53 Repression of c-Met Cooperation with p53 to repress c-Met	Dang et al., 2013
miR-181a	Inhibits cell motility and invasion	Inhabitation of c-Met	Korhan et al., 2014
miR-122	Induces apoptosis	Inhabitation of c-Met	Yang et al., 2015
miR-199	Represses cell motility, invasion, proliferation	Inhibition of c-Met downstream signaling Repression of CD44 and STAT3	Cooper et al., 1984; Henry et al., 2010
miR-26a	anti-angiogenesis	Inhibition of angiogenesis by suppressing VEGF-A and HGF Inhibition of c-Met downstream signaling Suppression of FBXO11 and ST3GAL5 signaling pathways	Yang et al., 2014; Cai et al., 2017; Ma et al., 2018
miR-148a	Promotes apoptosis, Suppress cell invasion	Suppression of EMT by targeting c-Met/SNAIL signaling	Zhang J.P. et al., 2014
miR-198	Inhibits cell migration, invasion	Inhibition of c-Met	Tan et al., 2011
miR-449	Represses cell migration, invasion Promotes apoptosis, Reduces proliferation	Inhibition of c-Met	Buurman et al., 2012
miR-93	Promotes cell proliferation, migration, invasion represses apoptosis	Activation of c-Met/P13K/AKT pathway Suppression of PTEN and CDKN1A Inhibition of PDCD4 and TIMP2	Ohta et al., 2015; Ji et al., 2017; Xue et al., 2018
miR-101	Suppresses cell migration, proliferation	Inhibition of c-Met Suppression of Girdin, SOX9, and TGF-β	Cao et al., 2016; Yang et al., 2016; Yan et al., 2018; Liu et al., 2019
miR-206	Suppresses proliferation, Induces apoptosis	Inhibition of c-Met	Wu et al., 2017

### Side Effect of c-Met Inhibitors

Although c-Met inhibitors show a survival benefit for advanced HCC, there has also been some toxicity and adverse effects demonstrated. For instance, compound 8 was a potent and highly selective ATP competitive c-Met inhibitor. Moreover, compound 8 showed good oral bioavailability and good half life and moderate plasma clearance and volume distribution. In addition, Compound 8 also demonstrated effective tumor inhibition. However, it increased heart rate, cardiac output, and induced myocardial degeneration in mice and thus was terminated as a preclinical candidate (Cui et al., 2013). Also, adverse effects such as hypertension, decreased appetite, ascites and pyrexia were found in a phase I/II multicenter study of the single-agent foretinib (Yau et al., 2017). Additionally, ascites, anemia, abdominal pain, and neutropenia were observed in a phase III study of tivantinib (Rimassa et al., 2018). The reason for these aforementioned side effects may be correlated with the physiological function of HGF/c-Met in many organs, including cytoprotective, regenerative, and reduction of apoptosis after injury (Birchmeier et al., 2003). Thus, c-Met inhibitors may block the physiological function of HGF/c-Met and induce side effects. Moreover, the adverse effect of c-Met inhibitors may be due to the inhibition of non-c-Met targets when using non-selective c-Met inhibitors.

### Inhibitors of c-Met Downstream Mediators

To solve the side effects mentioned above, several therapy strategies have been suggested. Among them, specifically targeting the downstream mediators of c-Met involved in tumor progression is a promising method, including the Grb2 SH2 domain, Src, MAPK, STAT3, Shp2 (Liu et al., 2018), and Fak (Jo et al., 2015). Nevertheless, there are still problems, especially because these signal pathways are shared with other RTKs, which may cause other unpredictable reactions. Therefore, this requires the identification of more specific and suitable downstream targets.

Recently endosomal processing has been demonstrated to play a pivotal role in the progression of HCC (Hu et al., 2015). Receptor endocytosis is crucial for signal transduction, either clathrin-dependent or –independent (Sorkin and von Zastrow, 2009; McMahon and Boucrot, 2011). HGF binding to c-Met induces the activation of downstream signal mediators, including ERK (You et al., 2011), c-Jun N-terminal kinase (JNK) (Rodrigues et al., 1997) and AKT (Zhang et al., 2018c). Both JNK and ERK mediate HCC cell migration by phosphorylating paxillin at serine residues, which is called an HGF-induced focal adhesion signaling molecule (Hu et al., 2015). Protein kinases C ε (PKCε) and golgi-localized γ-ear-containing ARF binding protein 3 (GGA3) regulate HGF-induced c-Met endocytosis (Kermorgant et al., 2004) to direct fluctuating JNK and paxillin signaling pathways which involve HCC cell migration (Hu et al., 2015). Importantly, endocytosis blockers, such as dynasore, could prevent the HGF-induced HCC cell migration and invasion by inhibiting critical endosomal components (Hu et al., 2015). Thus, critical endosomal components may be promising targets in HGF/c-Met signaling pathways for HCC treatment. In addition, Hu et al. (2017) suggested that hydrogen peroxide-inducible clone-5 (Hic-5) may be crucial for c-Met signaling pathways and HCC metastasis because it mediates HGF-induced reactive oxygen species (ROS)-JNK-signaling pathways in HCC (Wu et al., 2015) and may be also a specific and safe target for treating HCC patients.

### Natural Compound and Herbal Medicines

In recent years, more and more studies have found that natural compounds can inhibit the progression of liver cancer. For example, deguelin can suppress tumor angiogenesis on vascular endothelial cells by decreasing autocrine VEGF and repressing HGF-induced c-Met signaling pathways, thereby inhibiting HCC progression (Li et al., 2018). Also, Cinobufacini, a well-known traditional Chinese medicine extracted from toad skins and venom glands, has a therapeutic effect in HCC (Qi et al., 2018). A study has found that Cinobufacini could suppress HepG2 cell invasion and metastasis through the inhibition of the c-Met/ERK induced EMT (Qi et al., 2018). Moreover, madecassoside (MAD), isolated from *Centella asiatica* (Li et al., 2016) could repress the activation of the HGF-induced c-Met-PKC-ERK1/2-Cyclooxygenase-2 (COX-2)-Prostaglandin E2 (PGE2) cascade to inhibit HCC cell proliferation and invasion. In conclusion, natural compounds and herbals may be potential therapeutic targets for HCC.

### Resistance in c-Met Inhibitors and Sorafenib and Combined Inhibition of HGF/c-Met and Other Pathways

C-Met inhibitor therapy has failed to result in satisfactory outcomes in phase III clinical trials for HCC. Therefore, it is urgent to understand mechanisms and find new strategies such as effective combination therapies. Several studies have shown resistance in c-Met inhibitors due to various mechanisms. First, c-Met inhibitors only affect high-expression c-Met patients with HCC. Thus, enrolled low-expression c-Met patients with HCC could lead to the occurrence of resistance in c-Met inhibitors. Second, as mentioned above, c-Met inhibitors which target the interaction between HGF and c-Met may lose efficacy owing to cell attachment (Wang et al., 2001), gene amplification of c-Met, DCP binding to c-Met (Suzuki et al., 2005), gene mutation in the c-Met activation loop (Okuma and Kondo, 2016) and crosstalk with other membrane receptors (Garcia-Vilas and Medina, 2018). Third, inhibition of c-Met signaling pathways triggers the EGFR pathway as a compensatory survival pathway (Steinway et al., 2015). Fourth, phosphorylation status of FGFR determines different sensitivities of HCC cells to c-Met inhibitors (Jo et al., 2015). Fifth, Li H. et al. (2019) suggested that c-Met inhibitors up-regulate the expression of PD-L1 in HCC cells by suppressing GSK3B-mediated PD-L1 degradation and induce T-cell suppression and tumor evasion of the immune response. Finally, when inhibiting the activation of HGF-induced c-Met, HCC cells can sustain survival through Y1234/1235-dephosphorylated c-Met induced autophagy (Huang et al., 2019).

New therapeutic strategies have been developed against the mechanism of c-Met inhibitors resistance described above. Among them, combined c-Met inhibitors and other pathway inhibitors is a promising treatment. For example, combined inhibition of both c-Met and EGFR pathways could repress the tumor growth of HCC (Steinway et al., 2015). Additionally, targeting both c-Met and FGFR pathways provides superior suppression of HCC progression (Jo et al., 2015). Also, combination of c-Met inhibitor and anti-PD1 treatment represses HCC growth and improves mouse survival (Li H. et al., 2019). Moreover, targeting c-Met and autophagy could overcome resistance in HCC (Huang et al., 2019).

Recently, a growing body of evidence suggests that aberrant activation of HGF/c-Met signaling is associated with resistance of target therapies (Corso and Giordano, 2013), including sorafenib. Sorafenib is a standard therapy for advanced HCC, thus the resistance to sorafenib is a major concerning problem. Firtina Karagonlar et al. (2016) have found that in HCC patients on long term sorafenib treatment, the upregulation of HGF induces autocrine activation of HGF/c-Met signaling pathways, increasing the invasion and migration abilities of HCC cells and leading to resistance to sorafenib. Moreover, a recent study has demonstrated that tumor associated M2 macrophages secret HGF in a feed-forward manner, leading the resistance to sorafenib (Dong et al., 2019). Also, a new study found that HGF activates phosphorylated (P)-ERK/Snail/EMT, P-STAT3/Snail/EMT (Chen et al., 2019) and AKT/ERK1/2-EGR1 (Han et al., 2017; Chen and Xia, 2019; Xiang et al., 2019) signaling pathways to induce the resistance to sorafenib. According to the study of Chen, IncRNA NEAT1 induces sorafenib resistance of HCC patients through repressing miR-335 expression and activation of c-Met-Akt signaling pathway (Chen and Xia, 2019). Thus, the concentration of HGF in serum may be a potential predictive marker for sorafenib efficacy (Shao et al., 2015) and the combination of HGF and c-Met inhibitors and sorefenib could improve the efficacy of the first line systemic treatment (Goyal et al., 2013; Dong et al., 2019). For instance, regorafenib plays a crucial role of reversing HGF-induced sorafenib resistance through the inhibition of the EMT (Chen et al., 2016, 2019). Angiopoietin-like protein (ANGPTL1) not only inhibits sorafenib resistance, but also inhibits cancer stemness and tumor growth of HCC cells via suppressing the EMT through the Met-AKT/ERK-EGR-1-Slug signaling cascade (Chen et al., 2016).

In conclusion, although the tivantinib phase III trial failed and the reason is not clear, the role of c-Met inhibitors in treating HCC can not be denied. C-Met inhibitors are still mainstream of research and deserve more research on its cause of failure and new clinical trials. miRNAs, natural compound and herbal medicines are emerging treatments of HCC which can inhibit multiple pathways including c-Met signaling pathway. But it also means that there may be other side effects. The advantage of c-met downstream pathway inhibitors is that they are highly targeted and have little side effects, but this requires us to further understand the key targets of HCC. The drug resistance may be due to the heterogeneity of liver cancer cells and combination therapy may be a good solution.

## Conclusion

The HGF/c-Met axis has an important role in cellular behaviors such as cell proliferation, migration, survival, migration, morphogenesis, and the EMT. Moreover, it is also essential for liver formation, growth, regeneration and protection and angiogenesis during embryonic development and in adulthood after injury. Especially in chronic liver diseases, inflammation decreases hepatocytes and increases the need for c-Met activity to promote hepatocyte proliferation, regeneration and suppress inflammation. Nevertheless, the aberrant activation of c-Met and downstream signaling pathways through overexpression of HGF or c-Met, gene amplification, mutational activation of c-Met, down-regulation of Met-targeted miRNA, binding to other ligands, autocrine signaling or abnormally high HGF levels initiates and drives tumorigenesis and promotes tumor growth, invasion, metastasis, and angiogenesis in HCC. In the progression of liver cancer, c-Met is regulated by various factors such as miRNAs and SOCS1. Furthermore, c-Met cooperates with other signaling pathways such as MUCI or β-catenin in promoting tumorigenesis. Therefore, the treatment of liver cancer with c-Met as a target is a potential and promising therapy strategy. So far, six c-Met inhibitors have entered clinical trials and have been shown to inhibit tumor growth and invasion. Moreover, selective c-Met inhibitors are superior to non-selective c-Met inhibitors in the treatment of liver cancer due to their low toxicity. However, the failure of the tivantinib phase III trial suggests that we need to further study the causes of failure and the feasibility of c-Met inhibitors in treating HCC.

In addition, we should also consider the relationship between c-Met inhibitors and liver disease, although there is no clinical evidence that c-Met inhibitors worsen liver function. In chronic liver diseases, c-Met expression is increased to promote hepatocyte proliferation and inhibit inflammation. C-Met inhibitors suppress this positive regulation and may accelerate advanced liver disease. Meanwhile, liver diseases affect the drug pharmacokinetics, pharmacodynamics, reduce enzyme activity, impair hepatic clearance of drugs and even change the interplay between drugs. These effects may alter the dose of the drug needed to reach the desired blood concentration and induce novel toxicities. Thus, we should consider whether patients with Child-Pugh B or C disease can tolerate the dose established in clinical trials because in the past, most clinical trials were conducted in patients with Child-Pugh A disease.

Besides c-Met inhibitors, many new therapeutic strategies have been developed, such as the use of miRNAs to regulate HGF/c-Met signaling pathways to inhibit liver cancer progression, targeting endocytosis and more downstream molecules, Hic-5 as a therapeutic strategy to reduce side effects of c-Met inhibitors, as well as herbal treatments. c-Met is also involved in the resistance mechanism of sorafenib, which can be solved by the combination of c-Met inhibitor and sorafenib. For the self-resistance of c-Met inhibitors, the combination of c-Met inhibitors and other inhibitors can be used to solve this problem, such as FGFR and EGFT inhibitors, autophagy inhibitor and anti-PD1 treatment. Whether HGF/c-Met can be used as independent diagnostic and prognostic markers still requires further research, but HGF/c-Met in combination with other diagnostic and prognostic markers is valuable. In general, the mechanism of the HGF/c-Met pathway involvement in liver cancer requires more research, and c-Met inhibitors are a potential and promising therapeutic strategy in patients with HCC.

## Author Contributions

ZR and ZY proposed the study and the guarantors. HW, BR, and JL performed the research and wrote the first draft. All authors contributed to the interpretation of the study and to further drafts.

## Conflict of Interest

The authors declare that the research was conducted in the absence of any commercial or financial relationships that could be construed as a potential conflict of interest.

## References

[B1] Abella J. V. Peschard P. Naujokas M. A. Lin T. Saucier C. Urbe S. (2005). Met/Hepatocyte growth factor receptor ubiquitination suppresses transformation and is required for Hrs phosphorylation. *Mol. Cell Biol.* 25 9632–9645. 10.1128/mcb.25.21.9632-9645.2005 16227611 PMC1265818

[B2] Abounader R. Laterra J. (2005). Scatter factor/hepatocyte growth factor in brain tumor growth and angiogenesis. *Neuro Oncol.* 7 436–451. 10.1215/s1152851705000050 16212809 PMC1871724

[B3] Aebersold D. M. Landt O. Berthou S. Gruber G. Beer K. T. Greiner R. H. (2003). Prevalence and clinical impact of Met Y1253D-activating point mutation in radiotherapy-treated squamous cell cancer of the oropharynx. *Oncogene* 22 8519–8523. 10.1038/sj.onc.1206968 14627992

[B4] Aoyama A. Katayama R. Oh-Hara T. Sato S. Okuno Y. Fujita N. (2014). Tivantinib (ARQ 197) exhibits antitumor activity by directly interacting with tubulin and overcomes ABC transporter-mediated drug resistance. *Mol. Cancer Ther.* 13 2978–2990. 10.1158/1535-7163.Mct-14-0462 25313010

[B5] Basilico C. Hultberg A. Blanchetot C. de Jonge N. Festjens E. Hanssens V. (2014). Four individually druggable MET hotspots mediate HGF-driven tumor progression. *J. Clin. Invest.* 124 3172–3186. 10.1172/jci72316 24865428 PMC4071368

[B6] Birchmeier C. Birchmeier W. Gherardi E. Vande Woude G. F. (2003). Met, metastasis, motility and more. *Nat. Rev. Mol. Cell Biol.* 4 915–925. 10.1038/nrm1261 14685170

[B7] Bladt F. Friese-Hamim M. Ihling C. Wilm C. Blaukat A. (2014). The c-Met inhibitor MSC2156119J effectively inhibits tumor growth in liver cancer models. *Cancers* 6 1736–1752. 10.3390/cancers6031736 25256830 PMC4190565

[B8] Blagotinsek K. Rozman D. (2017). Targeting signalling pathways in Hepatocellular Carcinoma. *Curr. Pharm. Des.* 23 170–175. 10.2174/1381612822666161006160005 27719638

[B9] Boccaccio C. Comoglio P. M. (2006). Invasive growth: a MET-driven genetic programme for cancer and stem cells. *Nat. Rev. Cancer* 6 637–645. 10.1038/nrc1912 16862193

[B10] Borowiak M. Garratt A. N. Wustefeld T. Strehle M. Trautwein C. Birchmeier C. (2004). Met provides essential signals for liver regeneration. *Proc. Natl. Acad. Sci. U.S.A.* 101 10608–10613. 10.1073/pnas.0403412101 15249655 PMC490025

[B11] Bouattour M. Raymond E. Qin S. Cheng A. L. Stammberger U. Locatelli G. (2018). Recent developments of c-Met as a therapeutic target in hepatocellular carcinoma. *Hepatology* 67 1132–1149. 10.1002/hep.29496 28862760 PMC5873445

[B12] Bozkaya G. Korhan P. Cokakli M. Erdal E. Sagol O. Karademir S. (2012). Cooperative interaction of MUC1 with the HGF/c-Met pathway during hepatocarcinogenesis. *Mol. Cancer* 11:64. 10.1186/1476-4598-11-64 22962849 PMC3542123

[B13] Bradley C. A. Salto-Tellez M. Laurent-Puig P. Bardelli A. Rolfo C. Tabernero J. (2017). Targeting c-MET in gastrointestinal tumours: rationale, opportunities and challenges. *Nat. Rev. Clin. Oncol.* 14 562–576. 10.1038/nrclinonc.2017.40 28374784

[B14] Bruix J. Qin S. Merle P. Granito A. Huang Y. H. Bodoky G. (2017). Regorafenib for patients with hepatocellular carcinoma who progressed on sorafenib treatment (RESORCE): a randomised, double-blind, placebo-controlled, phase 3 trial. *Lancet* 389 56–66. 10.1016/s0140-6736(16)32453-32459 27932229

[B15] Buurman R. Gurlevik E. Schaffer V. Eilers M. Sandbothe M. Kreipe H. (2012). Histone deacetylases activate hepatocyte growth factor signaling by repressing microRNA-449 in hepatocellular carcinoma cells. *Gastroenterology* 143:e815. 10.1053/j.gastro.2012.05.033 22641068

[B16] Cai H. Zhou H. Miao Y. Li N. Zhao L. Jia L. (2017). MiRNA expression profiles reveal the involvement of miR-26a, miR-548l and miR-34a in hepatocellular carcinoma progression through regulation of ST3GAL5. *Lab. Invest.* 97 530–542. 10.1038/labinvest.2017.12 28218742

[B17] Cao K. Li J. Zhao Y. Wang Q. Zeng Q. He S. (2016). miR-101 inhibiting cell proliferation, migration and invasion in hepatocellular carcinoma through downregulating girdin. *Mol. Cells* 39 96–102. 10.14348/molcells.2016.2161 26743900 PMC4757808

[B18] Chang L. Hu Y. Fu Y. Zhou T. You J. Du J. (2019). Targeting slug-mediated non-canonical activation of c-Met to overcome chemo-resistance in metastatic ovarian cancer cells. *Acta Pharm. Sin. B* 9 484–495. 10.1016/j.apsb.2019.03.001 31193822 PMC6543058

[B19] Chen H. A. Kuo T. C. Tseng C. F. Ma J. T. Yang S. T. Yen C. J. (2016). Angiopoietin-like protein 1 antagonizes MET receptor activity to repress sorafenib resistance and cancer stemness in hepatocellular carcinoma. *Hepatology* 64 1637–1651. 10.1002/hep.28773 27530187

[B20] Chen S. Xia X. (2019). Long noncoding RNA NEAT1 suppresses sorafenib sensitivity of hepatocellular carcinoma cells via regulating miR-335-c-Met. *J. Cell Physiol.* 234 14999–15009. 10.1002/jcp.27567 30937906

[B21] Chen W. Yang J. Zhang Y. Cai H. Chen X. Sun D. (2019). Regorafenib reverses HGF-induced sorafenib resistance by inhibiting epithelial-mesenchymal transition in hepatocellular carcinoma. *FEBS Open Biol.* 9 335–347. 10.1002/2211-5463.12578 30761258 PMC6356182

[B22] Cheng A. L. Kang Y. K. Chen Z. Tsao C. J. Qin S. Kim J. S. (2009). Efficacy and safety of sorafenib in patients in the Asia-Pacific region with advanced hepatocellular carcinoma: a phase III randomised, double-blind, placebo-controlled trial. *Lancet Oncol.* 10 25–34. 10.1016/s1470-2045(08)70285-70287 19095497

[B23] Cooper C. S. Park M. Blair D. G. Tainsky M. A. Huebner K. Croce C. M. (1984). Molecular cloning of a new transforming gene from a chemically transformed human cell line. *Nature* 311 29–33. 10.1038/311029a0 6590967

[B24] Corso S. Giordano S. (2013). Cell-autonomous and non-cell-autonomous mechanisms of HGF/MET-driven resistance to targeted therapies: from basic research to a clinical perspective. *Cancer Discov.* 3 978–992. 10.1158/2159-8290.Cd-13-0040 23901039

[B25] Cui J. J. Shen H. Tran-Dube M. Nambu M. McTigue M. Grodsky N. (2013). Lessons from (S)-6-(1-(6-(1-methyl-1H-pyrazol-4-yl)-[1,2,4]triazolo[4,3-b]pyridazin-3-yl)ethyl)quinoline (PF-04254644), an inhibitor of receptor tyrosine kinase c-Met with high protein kinase selectivity but broad phosphodiesterase family inhibition leading to myocardial degeneration in rats. *J. Med. Chem.* 56 6651–6665. 10.1021/jm400926x 23944843

[B26] Dang Y. Luo D. Rong M. Chen G. (2013). Underexpression of miR-34a in hepatocellular carcinoma and its contribution towards enhancement of proliferating inhibitory effects of agents targeting c-MET. *PLoS One* 8:e61054. 10.1371/journal.pone.0061054 23593387 PMC3622605

[B27] Di Renzo M. F. Olivero M. Martone T. Maffe A. Maggiora P. Stefani A. D. (2000). Somatic mutations of the MET oncogene are selected during metastatic spread of human HNSC carcinomas. *Oncogene* 19 1547–1555. 10.1038/sj.onc.1203455 10734314

[B28] Ding W. You H. Dang H. LeBlanc F. Galicia V. Lu S. C. (2010). Epithelial-to-mesenchymal transition of murine liver tumor cells promotes invasion. *Hepatology* 52 945–953. 10.1002/hep.23748 20564331 PMC3032356

[B29] Dong N. Shi X. Wang S. Gao Y. Kuang Z. Xie Q. (2019). M2 macrophages mediate sorafenib resistance by secreting HGF in a feed-forward manner in hepatocellular carcinoma. *Br. J. Cancer* 121 22–33. 10.1038/s41416-019-0482-x 31130723 PMC6738111

[B30] Du Z. Caenepeel S. Shen Y. Rex K. Zhang Y. He Y. (2016). Preclinical evaluation of AMG 337, a highly selective small molecule MET inhibitor, in hepatocellular carcinoma. *Mol. Cancer Ther.* 15 1227–1237. 10.1158/1535-7163.Mct-15-0745 27196749

[B31] Dyson J. Jaques B. Chattopadyhay D. Lochan R. Graham J. Das D. (2014). Hepatocellular cancer: the impact of obesity, type 2 diabetes and a multidisciplinary team. *J. Hepatol.* 60 110–117. 10.1016/j.jhep.2013.08.011 23978719

[B32] El-Khoueiry A. B. Sangro B. Yau T. Crocenzi T. S. Kudo M. Hsu C. (2017). Nivolumab in patients with advanced hepatocellular carcinoma (CheckMate 040): an open-label, non-comparative, phase 1/2 dose escalation and expansion trial. *Lancet* 389 2492–2502. 10.1016/s0140-6736(17)31046-31042 28434648 PMC7539326

[B33] Fasolo A. Sessa C. Gianni L. Broggini M. (2013). Seminars in clinical pharmacology: an introduction to MET inhibitors for the medical oncologist. *Ann. Oncol.* 24 14–20. 10.1093/annonc/mds520 23110808

[B34] Ferlay J. Colombet M. Soerjomataram I. Mathers C. Parkin D. M. Pineros M. (2019). Estimating the global cancer incidence and mortality in 2018: GLOBOCAN sources and methods. *Int. J. Cancer* 144 1941–1953. 10.1002/ijc.31937 30350310

[B35] Firtina Karagonlar Z. Koc D. Iscan E. Erdal E. Atabey N. (2016). Elevated hepatocyte growth factor expression as an autocrine c-Met activation mechanism in acquired resistance to sorafenib in hepatocellular carcinoma cells. *Cancer Sci.* 107 407–416. 10.1111/cas.12891 26790028 PMC4832867

[B36] Folkman J. (2003). Fundamental concepts of the angiogenic process. *Curr. Mol. Med.* 3 643–651. 10.2174/1566524033479465 14601638

[B37] Forner A. Reig M. Bruix J. (2018). Hepatocellular carcinoma. *Lancet* 391 1301–1314. 10.1016/s0140-6736(18)30010-30012 29307467

[B38] Friedman R. C. Farh K. K. Burge C. B. Bartel D. P. (2009). Most mammalian mRNAs are conserved targets of microRNAs. *Genome Res.* 19 92–105. 10.1101/gr.082701.108 18955434 PMC2612969

[B39] Gao F. Deng G. Liu W. Zhou K. Li M. (2017). Resveratrol suppresses human hepatocellular carcinoma via targeting HGF-c-Met signaling pathway. *Oncol. Rep.* 37 1203–1211. 10.3892/or.2017.5347 28075467

[B40] Gao J. Inagaki Y. Song P. Qu X. Kokudo N. Tang W. (2012). Targeting c-Met as a promising strategy for the treatment of hepatocellular carcinoma. *Pharmacol. Res.* 65 23–30. 10.1016/j.phrs.2011.11.011 22138044

[B41] Garcia-Vilas J. A. Medina M. A. (2018). Updates on the hepatocyte growth factor/c-Met axis in hepatocellular carcinoma and its therapeutic implications. *World J. Gastroenterol.* 24 3695–3708. 10.3748/wjg.v24.i33.3695 30197476 PMC6127652

[B42] Ghiso E. Giordano S. (2013). Targeting MET: why, where and how? *Curr. Opin. Pharmacol.* 13 511–518. 10.1016/j.coph.2013.05.018 23797036

[B43] Giordano S. Columbano A. (2014). Met as a therapeutic target in HCC: facts and hopes. *J. Hepatol.* 60 442–452. 10.1016/j.jhep.2013.09.009 24045150

[B44] Gong X. Y. Ma N. Xu H. X. Chen F. Huang X. H. Wang Q. (2018). Prognostic significance of c-Met, beta-catenin and FAK in patients with hepatocellular carcinoma following surgery. *Oncol. Lett.* 15 3796–3805. 10.3892/ol.2018.7733 29467897 PMC5796308

[B45] Gonzalez M. N. de Mello W. Butler-Browne G. S. Silva-Barbosa S. D. Mouly V. Savino W. (2017). HGF potentiates extracellular matrix-driven migration of human myoblasts: involvement of matrix metalloproteinases and MAPK/ERK pathway. *Skelet Muscle* 7:20. 10.1186/s13395-017-0138-136 29017538 PMC5635537

[B46] Goyal L. Muzumdar M. D. Zhu A. X. (2013). Targeting the HGF/c-MET pathway in hepatocellular carcinoma. *Clin. Cancer Res.* 19 2310–2318. 10.1158/1078-0432.Ccr-12-2791 23388504 PMC4583193

[B47] Graveel C. R. Tolbert D. Vande Woude G. F. (2013). MET: a critical player in tumorigenesis and therapeutic target. *Cold Spring Harb. Perspect. Biol.* 5:a009209. 10.1101/cshperspect.a009209 23818496 PMC3685898

[B48] Gui Y. Khan M. G. M. Bobbala D. Dubois C. Ramanathan S. Saucier C. (2017). Attenuation of MET-mediated migration and invasion in hepatocellular carcinoma cells by SOCS1. *World J. Gastroenterol.* 23 6639–6649. 10.3748/wjg.v23.i36.6639 29085209 PMC5643285

[B49] Gui Y. Yeganeh M. Donates Y. C. Tobelaim W. S. Chababi W. Mayhue M. (2015). Regulation of MET receptor tyrosine kinase signaling by suppressor of cytokine signaling 1 in hepatocellular carcinoma. *Oncogene* 34 5718–5728. 10.1038/onc.2015.20 25728680

[B50] Gui Y. Yeganeh M. Ramanathan S. Leblanc C. Pomerleau V. Ferbeyre G. (2011). SOCS1 controls liver regeneration by regulating HGF signaling in hepatocytes. *J. Hepatol.* 55 1300–1308. 10.1016/j.jhep.2011.03.027 21703184

[B51] Han P. Li H. Jiang X. Zhai B. Tan G. Zhao D. (2017). Dual inhibition of Akt and c-Met as a second-line therapy following acquired resistance to sorafenib in hepatocellular carcinoma cells. *Mol. Oncol.* 11 320–334. 10.1002/1878-0261.12039 28164434 PMC5527443

[B52] He M. Peng A. Huang X. Z. Shi D. C. Wang J. C. Zhao Q. (2016). Peritumoral stromal neutrophils are essential for c-Met-elicited metastasis in human hepatocellular carcinoma. *Oncoimmunology* 5:e1219828. 10.1080/2162402x.2016.1219828 27853643 PMC5087290

[B53] Henry J. C. Park J. K. Jiang J. Kim J. H. Nagorney D. M. Roberts L. R. (2010). miR-199a-3p targets CD44 and reduces proliferation of CD44 positive hepatocellular carcinoma cell lines. *Biochem. Biophys. Res. Commun.* 403 120–125. 10.1016/j.bbrc.2010.10.130 21055388 PMC3039123

[B54] Hu C. T. Cheng C. C. Wu J. R. Pan S. M. Wu W. S. (2015). PKCepsilon-mediated c-Met endosomal processing directs fluctuant c-Met-JNK-paxillin signaling for tumor progression of HepG2. *Cell Signal.* 27 1544–1555. 10.1016/j.cellsig.2015.02.031 25778903

[B55] Hu C. T. Wu J. R. Cheng C. C. Wu W. S. (2017). The therapeutic targeting of HGF/c-Met signaling in hepatocellular carcinoma: alternative approaches. *Cancers* 9:58. 10.3390/cancers9060058 28587113 PMC5483877

[B56] Huang X. Gan G. Wang X. Xu T. Xie W. (2019). The HGF-MET axis coordinates liver cancer metabolism and autophagy for chemotherapeutic resistance. *Autophagy* 15 1258–1279. 10.1080/15548627.2019.1580105 30786811 PMC6613896

[B57] Janevska D. Chaloska-Ivanova V. Janevski V. (2015). Hepatocellular carcinoma: risk factors, diagnosis and treatment. *Open Access. Maced. J. Med. Sci.* 3 732–736. 10.3889/oamjms.2015.111 27275318 PMC4877918

[B58] Ji C. Liu H. Yin Q. Li H. Gao H. (2017). miR-93 enhances hepatocellular carcinoma invasion and metastasis by EMT via targeting PDCD4. *Biotechnol. Lett.* 39 1621–1629. 10.1007/s10529-017-2403-2405 28748353

[B59] Jia C. C. Wang T. T. Liu W. Fu B. S. Hua X. Wang G. Y. (2013). Cancer-associated fibroblasts from hepatocellular carcinoma promote malignant cell proliferation by HGF secretion. *PLoS One* 8:e63243. 10.1371/journal.pone.0063243 23667593 PMC3647063

[B60] Jo J. C. Choi E. K. Shin J. S. Moon J. H. Hong S. W. Lee H. R. (2015). Targeting FGFR pathway in human hepatocellular carcinoma: expressing pFGFR and pMET for antitumor activity. *Mol. Cancer Ther.* 14 2613–2622. 10.1158/1535-7163.Mct-14-0780 26351320

[B61] Junbo H. Li Q. Zaide W. Yunde H. (1999). Increased level of serum hepatocyte growth factor/scatter factor in liver cancer is associated with tumor metastasis. *In Vivo* 13 177–180. 10363175

[B62] Kang Y. K. Yau T. Park J. W. Lim H. Y. Lee T. Y. Obi S. (2015). Randomized phase II study of axitinib versus placebo plus best supportive care in second-line treatment of advanced hepatocellular carcinoma. *Ann. Oncol.* 26 2457–2463. 10.1093/annonc/mdv388 26386123

[B63] Kaposi-Novak P. Lee J. S. Gomez-Quiroz L. Coulouarn C. Factor V. M. Thorgeirsson S. S. (2006). Met-regulated expression signature defines a subset of human hepatocellular carcinomas with poor prognosis and aggressive phenotype. *J. Clin. Invest.* 116 1582–1595. 10.1172/jci27236 16710476 PMC1462944

[B64] Karabulut S. Tas F. Akyuz F. Ormeci A. C. Serilmez M. Soydinc H. O. (2014). Clinical significance of serum hepatocyte growth factor (HGF) levels in hepatocellular carcinoma. *Tumour Biol.* 35 2327–2333. 10.1007/s13277-013-1308-1308 24142532

[B65] Karagonlar Z. F. Korhan P. Atabey N. (2015). Targeting c-met in cancer by MicroRNAs: potential therapeutic applications in hepatocellular carcinoma. *Drug Dev. Res.* 76 357–367. 10.1002/ddr.21274 26363180

[B66] Ke A. W. Shi G. M. Zhou J. Wu F. Z. Ding Z. B. Hu M. Y. (2009). Role of overexpression of CD151 and/or c-Met in predicting prognosis of hepatocellular carcinoma. *Hepatology* 49 491–503. 10.1002/hep.22639 19065669

[B67] Kentsis A. Reed C. Rice K. L. Sanda T. Rodig S. J. Tholouli E. (2012). Autocrine activation of the MET receptor tyrosine kinase in acute myeloid leukemia. *Nat. Med.* 18 1118–1122. 10.1038/nm.2819 22683780 PMC3438345

[B68] Kermorgant S. Zicha D. Parker P. J. (2004). PKC controls HGF-dependent c-Met traffic, signalling and cell migration. *EMBO J.* 23 3721–3734. 10.1038/sj.emboj.7600396 15385963 PMC522795

[B69] Kondo S. Ojima H. Tsuda H. Hashimoto J. Morizane C. Ikeda M. (2013). Clinical impact of c-Met expression and its gene amplification in hepatocellular carcinoma. *Int. J. Clin. Oncol.* 18 207–213. 10.1007/s10147-011-0361-369 22218908

[B70] Korhan P. Erdal E. Atabey N. (2014). MiR-181a-5p is downregulated in hepatocellular carcinoma and suppresses motility, invasion and branching-morphogenesis by directly targeting c-Met. *Biochem. Biophys. Res. Commun.* 450 1304–1312. 10.1016/j.bbrc.2014.06.142 25058462

[B71] Kudo M. Finn R. S. Qin S. Han K. H. Ikeda K. Piscaglia F. (2018). Lenvatinib versus sorafenib in first-line treatment of patients with unresectable hepatocellular carcinoma: a randomised phase 3 non-inferiority trial. *Lancet* 391 1163–1173. 10.1016/s0140-6736(18)30207-30201 29433850

[B72] Kudo M. Han G. Finn R. S. Poon R. T. Blanc J. F. Yan L. (2014). Brivanib as adjuvant therapy to transarterial chemoembolization in patients with hepatocellular carcinoma: a randomized phase III trial. *Hepatology* 60 1697–1707. 10.1002/hep.27290 24996197

[B73] Ladeiro Y. Couchy G. Balabaud C. Bioulac-Sage P. Pelletier L. Rebouissou S. (2008). MicroRNA profiling in hepatocellular tumors is associated with clinical features and oncogene/tumor suppressor gene mutations. *Hepatology* 47 1955–1963. 10.1002/hep.22256 18433021

[B74] Lee J. H. Han S. U. Cho H. Jennings B. Gerrard B. Dean M. (2000). A novel germ line juxtamembrane Met mutation in human gastric cancer. *Oncogene* 19 4947–4953. 10.1038/sj.onc.1203874 11042681

[B75] Lee K. H. Hyun M. S. Kim J. R. (2003). Growth factor-dependent activation of the MAPK pathway in human pancreatic cancer: MEK/ERK and p38 MAP kinase interaction in uPA synthesis. *Clin. Exp. Metastasis* 20 499–505. 14598883 10.1023/a:1025824816021

[B76] Li H. Li C. W. Li X. Ding Q. Guo L. Liu S. (2019). MET inhibitors promote liver tumor evasion of the immune response by stabilizing PDL1. *Gastroenterology* 156:1849-1861.e13. 10.1053/j.gastro.2019.01.252 30711629 PMC6904924

[B77] Li J. Liu L. Liu X. Xu P. Hu Q. Yu Y. (2019). The Role of upregulated DDX11 as a potential prognostic and diagnostic biomarker in lung adenocarcinoma. *J. Cancer* 10 4208–4216. 10.7150/jca.33457 31413739 PMC6691710

[B78] Li M. Yu X. Li W. Liu T. Deng G. Liu W. (2018). Deguelin suppresses angiogenesis in human hepatocellular carcinoma by targeting HGF-c-Met pathway. *Oncotarget* 9 152–166. 10.18632/oncotarget.22077 29416603 PMC5787453

[B79] Li Z. You K. Li J. Wang Y. Xu H. Gao B. (2016). Madecassoside suppresses proliferation and invasiveness of HGF-induced human hepatocellular carcinoma cells via PKC-cMET-ERK1/2-COX-2-PGE2 pathway. *Int. Immunopharmacol.* 33 24–32. 10.1016/j.intimp.2016.01.027 26851630

[B80] Liang Y. Feng Y. Zong M. Wei X. F. Lee J. Feng Y. (2018). beta-catenin deficiency in hepatocytes aggravates hepatocarcinogenesis driven by oncogenic beta-catenin and MET. *Hepatology* 67 1807–1822. 10.1002/hep.29661 29152756 PMC5906147

[B81] Liotta L. A. Kohn E. C. (2001). The microenvironment of the tumour-host interface. *Nature* 411 375–379. 10.1038/35077241 11357145

[B82] Liu J. J. Li Y. Chen W. S. Liang Y. Wang G. Zong M. (2018). Shp2 deletion in hepatocytes suppresses hepatocarcinogenesis driven by oncogenic beta-Catenin, PIK3CA and MET. *J. Hepatol.* 69 79–88. 10.1016/j.jhep.2018.02.014 29505847 PMC6008184

[B83] Liu W. T. Jing Y. Y. Yu G. F. Chen H. Han Z. P. Yu D. D. (2016). Hepatic stellate cell promoted hepatoma cell invasion via the HGF/c-Met signaling pathway regulated by p53. *Cell Cycle* 15 886–894. 10.1080/15384101.2016.1152428 27077227 PMC4889302

[B84] Liu Y. Tan J. Ou S. Chen J. Chen L. (2019). MicroRNA-101-3p suppresses proliferation and migration in hepatocellular carcinoma by targeting the HGF/c-Met pathway. *Invest. New Drugs* 10.1007/s10637-019-00766-768 [Epub ahead of print] 30929159

[B85] Llovet J. M. Ricci S. Mazzaferro V. Hilgard P. Gane E. Blanc J. F. (2008). Sorafenib in advanced hepatocellular carcinoma. *N. Engl. J. Med.* 359 378–390. 10.1056/NEJMoa0708857 18650514

[B86] Llovet J. M. Villanueva A. Lachenmayer A. Finn R. S. (2015). Advances in targeted therapies for hepatocellular carcinoma in the genomic era. *Nat. Rev. Clin. Oncol.* 12:436. 10.1038/nrclinonc.2015.121 26099984

[B87] Luo T. Zhang S. G. Zhu L. F. Zhang F. X. Li W. Zhao K. (2019). A selective c-Met and Trks inhibitor Indo5 suppresses hepatocellular carcinoma growth. *J. Exp. Clin. Cancer Res.* 38:130. 10.1186/s13046-019-1104-1104 30885237 PMC6421704

[B88] Ma Y. Deng F. Li P. Chen G. Tao Y. Wang H. (2018). The tumor suppressive miR-26a regulation of FBXO11 inhibits proliferation, migration and invasion of hepatocellular carcinoma cells. *Biomed. Pharmacother.* 101 648–655. 10.1016/j.biopha.2018.02.118 29518611

[B89] Marisi G. Petracci E. Raimondi F. Faloppi L. Foschi F. G. Lauletta G. (2019). ANGPT2 and NOS3 polymorphisms and clinical outcome in advanced hepatocellular carcinoma patients receiving sorafenib. *Cancers* 11:E1023. 10.3390/cancers11071023 31330833 PMC6679015

[B90] Maroun C. R. Rowlands T. (2014). The Met receptor tyrosine kinase: a key player in oncogenesis and drug resistance. *Pharmacol. Ther.* 142 316–338. 10.1016/j.pharmthera.2013.12.014 24384534

[B91] Marquardt J. U. Thorgeirsson S. S. (2013). Linking MLL and the HGF-MET signaling pathway in liver cancer. *J. Clin. Invest.* 123 2780–2783. 10.1172/jci70235 23934122 PMC3696572

[B92] Marx-Stoelting P. Borowiak M. Knorpp T. Birchmeier C. Buchmann A. Schwarz M. (2009). Hepatocarcinogenesis in mice with a conditional knockout of the hepatocyte growth factor receptor c-Met. *Int. J. Cancer* 124 1767–1772. 10.1002/ijc.24167 19123478

[B93] Mas V. R. Maluf D. G. Archer K. J. Yanek K. C. Fisher R. A. (2007). Angiogenesis soluble factors as hepatocellular carcinoma noninvasive markers for monitoring hepatitis C virus cirrhotic patients awaiting liver transplantation. *Transplantation* 84 1262–1271. 10.1097/01.tp.0000287596.91520.1a 18049111

[B94] Matsumoto K. Nakamura T. Sakai K. Nakamura T. (2008). Hepatocyte growth factor and Met in tumor biology and therapeutic approach with NK4. *Proteomics* 8 3360–3370. 10.1002/pmic.200800156 18646008

[B95] McMahon H. T. Boucrot E. (2011). Molecular mechanism and physiological functions of clathrin-mediated endocytosis. *Nat. Rev. Mol. Cell Biol.* 12 517–533. 10.1038/nrm3151 21779028

[B96] Migliore C. Giordano S. (2008). Molecular cancer therapy: can our expectation be MET? *Eur. J. Cancer* 44 641–651. 10.1016/j.ejca.2008.01.022 18295476

[B97] Munshi N. Jeay S. Li Y. Chen C. R. France D. S. Ashwell M. A. (2010). ARQ 197, a novel and selective inhibitor of the human c-Met receptor tyrosine kinase with antitumor activity. *Mol. Cancer Ther.* 9 1544–1553. 10.1158/1535-7163.Mct-09-1173 20484018

[B98] Murakami Y. Yasuda T. Saigo K. Urashima T. Toyoda H. Okanoue T. (2006). Comprehensive analysis of microRNA expression patterns in hepatocellular carcinoma and non-tumorous tissues. *Oncogene* 25 2537–2545. 10.1038/sj.onc.1209283 16331254

[B99] Musso O. Beraza N. (2019). Hepatocellular carcinomas: evolution to sorafenib resistance through hepatic leukaemia factor. *Gut* 68 1728–1730. 10.1136/gutjnl-2019-318999 31270163 PMC6839724

[B100] Nakamura T. Nawa K. Ichihara A. (1984). Partial purification and characterization of hepatocyte growth factor from serum of hepatectomized rats. *Biochem. Biophys. Res. Commun.* 122 1450–1459. 10.1016/0006-291x(84)91253-91251 6477569

[B101] Ohta K. Hoshino H. Wang J. Ono S. Iida Y. Hata K. (2015). MicroRNA-93 activates c-Met/PI3K/Akt pathway activity in hepatocellular carcinoma by directly inhibiting PTEN and CDKN1A. *Oncotarget* 6 3211–3224. 10.18632/oncotarget.3085 25633810 PMC4413648

[B102] Okuma H. S. Kondo S. (2016). Trends in the development of MET inhibitors for hepatocellular carcinoma. *Future Oncol.* 12 1275–1286. 10.2217/fon.16.3 26984595

[B103] Park W. S. Dong S. M. Kim S. Y. Na E. Y. Shin M. S. Pi J. H. (1999). Somatic mutations in the kinase domain of the Met/hepatocyte growth factor receptor gene in childhood hepatocellular carcinomas. *Cancer Res.* 59 307–310. 9927037

[B104] Pascale R. M. Feo F. Calvisi D. F. (2016). An infernal cross-talk between oncogenic beta-catenin and c-Met in hepatocellular carcinoma: evidence from mouse modeling. *Hepatology* 64 1421–1423. 10.1002/hep.28790 27596836

[B105] Pennacchietti S. Michieli P. Galluzzo M. Mazzone M. Giordano S. Comoglio P. M. (2003). Hypoxia promotes invasive growth by transcriptional activation of the met protooncogene. *Cancer Cell* 3 347–361. 12726861 10.1016/s1535-6108(03)00085-0

[B106] Peschard P. Park M. (2007). From Tpr-Met to Met, tumorigenesis and tubes. *Oncogene* 26 1276–1285. 10.1038/sj.onc.1210201 17322912

[B107] Qi F. Wang J. Zhao L. Cai P. Tang W. Wang Z. (2018). Cinobufacini inhibits epithelial-mesenchymal transition of human hepatocellular carcinoma cells through c-Met/ERK signaling pathway. *Biosci. Trends* 12 291–297. 10.5582/bst.2018.01082 29794405

[B108] Qiao Y. Wang J. Karagoz E. Liang B. Song X. Shang R. (2019). Axis inhibition protein 1 (Axin1) deletion-induced hepatocarcinogenesis requires intact beta-catenin but not notch cascade in mice. *Hepatology* 70 2003–2017. 10.1002/hep.30556 30737831 PMC7206928

[B109] Remsing Rix L. L. Kuenzi B. M. Luo Y. Remily-Wood E. Kinose F. Wright G. (2014). GSK3 alpha and beta are new functionally relevant targets of tivantinib in lung cancer cells. *ACS Chem. Biol.* 9 353–358. 10.1021/cb400660a 24215125 PMC3944088

[B110] Ren Z. Chen X. Hong L. Zhao X. Cui G. Li A. (2020). Nanoparticle conjugation of ginsenoside Rg3 inhibits hepatocellular carcinoma development and metastasis. *Small* 16:e1905233. 10.1002/smll.201905233 31814271

[B111] Rimassa L. Assenat E. Peck-Radosavljevic M. Pracht M. Zagonel V. Mathurin P. (2018). Tivantinib for second-line treatment of MET-high, advanced hepatocellular carcinoma (METIV-HCC): a final analysis of a phase 3, randomised, placebo-controlled study. *Lancet Oncol.* 19 682–693. 10.1016/s1470-2045(18)30146-30143 29625879

[B112] Rodrigues G. A. Park M. Schlessinger J. (1997). Activation of the JNK pathway is essential for transformation by the Met oncogene. *EMBO J.* 16 2634–2645. 10.1093/emboj/16.10.2634 9184210 PMC1169874

[B113] Santoro A. Rimassa L. Borbath I. Daniele B. Salvagni S. Van Laethem J. L. (2013). Tivantinib for second-line treatment of advanced hepatocellular carcinoma: a randomised, placebo-controlled phase 2 study. *Lancet Oncol.* 14 55–63. 10.1016/s1470-2045(12)70490-70494 23182627

[B114] Saucier C. Khoury H. Lai K. M. Peschard P. Dankort D. Naujokas M. A. (2004). The Shc adaptor protein is critical for VEGF induction by Met/HGF and ErbB2 receptors and for early onset of tumor angiogenesis. *Proc. Natl. Acad. Sci. U.S.A.* 101 2345–2350. 10.1073/pnas.0308065101 14983012 PMC356953

[B115] Scagliotti G. V. Novello S. von Pawel J. (2013). The emerging role of MET/HGF inhibitors in oncology. *Cancer Treat. Rev.* 39 793–801. 10.1016/j.ctrv.2013.02.001 23453860

[B116] Schiffer E. Housset C. Cacheux W. Wendum D. Desbois-Mouthon C. Rey C. (2005). Gefitinib, an EGFR inhibitor, prevents hepatocellular carcinoma development in the rat liver with cirrhosis. *Hepatology* 41 307–314. 10.1002/hep.20538 15660382

[B117] Schmidt C. Bladt F. Goedecke S. Brinkmann V. Zschiesche W. Sharpe M. (1995). Scatter factor/hepatocyte growth factor is essential for liver development. *Nature* 373 699–702. 10.1038/373699a0 7854452

[B118] Schmidt L. Junker K. Nakaigawa N. Kinjerski T. Weirich G. Miller M. (1999). Novel mutations of the MET proto-oncogene in papillary renal carcinomas. *Oncogene* 18 2343–2350. 10.1038/sj.onc.1202547 10327054

[B119] Semela D. Dufour J. F. (2004). Angiogenesis and hepatocellular carcinoma. *J. Hepatol.* 41 864–880. 10.1016/j.jhep.2004.09.006 15519663

[B120] Shao Y. Y. Hsu C. H. Cheng A. L. (2015). Predictive biomarkers of sorafenib efficacy in advanced hepatocellular carcinoma: are we getting there? *World J. Gastroenterol.* 21 10336–10347. 10.3748/wjg.v21.i36.10336 26420960 PMC4579880

[B121] Sorkin A. von Zastrow M. (2009). Endocytosis and signalling: intertwining molecular networks. *Nat. Rev. Mol. Cell Biol.* 10 609–622. 10.1038/nrm2748 19696798 PMC2895425

[B122] Steinway S. N. Dang H. You H. Rountree C. B. Ding W. (2015). The EGFR/ErbB3 pathway acts as a compensatory survival mechanism upon c-Met inhibition in human c-Met+ hepatocellular carcinoma. *PLoS One* 10:e0128159. 10.1371/journal.pone.0128159 26000702 PMC4441360

[B123] Stoker M. Gherardi E. Perryman M. Gray J. (1987). Scatter factor is a fibroblast-derived modulator of epithelial cell mobility. *Nature* 327 239–242. 10.1038/327239a0 2952888

[B124] Sun C. Y. Zhu Y. Li X. F. Tang L. P. Su Z. Q. Wang X. Q. (2017). Norcantharidin alone or in combination with crizotinib induces autophagic cell death in hepatocellular carcinoma by repressing c-Met-mTOR signaling. *Oncotarget* 8 114945–114955. 10.18632/oncotarget.22935 29383132 PMC5777744

[B125] Suzuki M. Shiraha H. Fujikawa T. Takaoka N. Ueda N. Nakanishi Y. (2005). Des-gamma-carboxy prothrombin is a potential autologous growth factor for hepatocellular carcinoma. *J. Biol. Chem.* 280 6409–6415. 10.1074/jbc.M406714200 15582995

[B126] Taher T. E. Derksen P. W. de Boer O. J. Spaargaren M. Teeling P. van der Wal A. C. (2002). Hepatocyte growth factor triggers signaling cascades mediating vascular smooth muscle cell migration. *Biochem. Biophys. Res. Commun.* 298 80–86. 10.1016/s0006-291x(02)02397-2395 12379223

[B127] Takami T. Kaposi-Novak P. Uchida K. Gomez-Quiroz L. E. Conner E. A. Factor V. M. (2007). Loss of hepatocyte growth factor/c-Met signaling pathway accelerates early stages of N-nitrosodiethylamine induced hepatocarcinogenesis. *Cancer Res.* 67 9844–9851. 10.1158/0008-5472.Can-07-1905 17942915

[B128] Takeda S. Liu H. Sasagawa S. Dong Y. Trainor P. A. Cheng E. H. (2013). HGF-MET signals via the MLL-ETS2 complex in hepatocellular carcinoma. *J. Clin. Invest.* 123 3154–3165. 10.1172/jci65566 23934123 PMC3696564

[B129] Takeo S. Arai H. Kusano N. Harada T. Furuya T. Kawauchi S. (2001). Examination of oncogene amplification by genomic DNA microarray in hepatocellular carcinomas: comparison with comparative genomic hybridization analysis. *Cancer Genet. Cytogenet.* 130 127–132. 10.1016/s0165-4608(01)00479-474 11675133

[B130] Tan S. Li R. Ding K. Lobie P. E. Zhu T. (2011). miR-198 inhibits migration and invasion of hepatocellular carcinoma cells by targeting the HGF/c-MET pathway. *FEBS Lett.* 585 2229–2234. 10.1016/j.febslet.2011.05.042 21658389

[B131] Tao J. Xu E. Zhao Y. Singh S. Li X. Couchy G. (2016). Modeling a human hepatocellular carcinoma subset in mice through coexpression of met and point-mutant beta-catenin. *Hepatology* 64 1587–1605. 10.1002/hep.28601 27097116 PMC5073058

[B132] Tavian D. De Petro G. Benetti A. Portolani N. Giulini S. M. Barlati S. (2000). u-PA and c-MET mRNA expression is co-ordinately enhanced while hepatocyte growth factor mRNA is down-regulated in human hepatocellular carcinoma. *Int. J. Cancer* 87 644–649. 10925356

[B133] Ueki T. Fujimoto J. Suzuki T. Yamamoto H. Okamoto E. (1997). Expression of hepatocyte growth factor and its receptor c-met proto-oncogene in hepatocellular carcinoma. *Hepatology* 25 862–866. 10.1002/hep.510250413 9096589

[B134] Unic A. Derek L. Duvnjak M. Patrlj L. Rakic M. Kujundzic M. (2018). Diagnostic specificity and sensitivity of PIVKAII, GP3, CSTB, SCCA1 and HGF for the diagnosis of hepatocellular carcinoma in patients with alcoholic liver cirrhosis. *Ann. Clin. Biochem.* 55 355–362. 10.1177/0004563217726808 28766361

[B135] Vejchapipat P. Tangkijvanich P. Theamboonlers A. Chongsrisawat V. Chittmittrapap S. Poovorawan Y. (2004). Association between serum hepatocyte growth factor and survival in untreated hepatocellular carcinoma. *J. Gastroenterol.* 39 1182–1188. 10.1007/s00535-004-1469-1468 15622483

[B136] Volinia S. Calin G. A. Liu C. G. Ambs S. Cimmino A. Petrocca F. (2006). A microRNA expression signature of human solid tumors defines cancer gene targets. *Proc. Natl. Acad. Sci. U.S.A.* 103 2257–2261. 10.1073/pnas.0510565103 16461460 PMC1413718

[B137] Wang R. Ferrell L. D. Faouzi S. Maher J. J. Bishop J. M. (2001). Activation of the Met receptor by cell attachment induces and sustains hepatocellular carcinomas in transgenic mice. *J. Cell Biol.* 153 1023–1034. 10.1083/jcb.153.5.1023 11381087 PMC2174327

[B138] Wang S. W. Pan S. L. Peng C. Y. Huang D. Y. Tsai A. C. Chang Y. L. (2007). CHM-1 inhibits hepatocyte growth factor-induced invasion of SK-Hep-1 human hepatocellular carcinoma cells by suppressing matrix metalloproteinase-9 expression. *Cancer Lett.* 257 87–96. 10.1016/j.canlet.2007.07.002 17689859

[B139] Whittaker S. Marais R. Zhu A. X. (2010). The role of signaling pathways in the development and treatment of hepatocellular carcinoma. *Oncogene* 29 4989–5005. 10.1038/onc.2010.236 20639898

[B140] Wilhelm S. Carter C. Lynch M. Lowinger T. Dumas J. Smith R. A. (2006). Discovery and development of sorafenib: a multikinase inhibitor for treating cancer. *Nat. Rev. Drug Discov.* 5 835–844. 10.1038/nrd2130 17016424

[B141] Williams R. Aspinall R. Bellis M. Camps-Walsh G. Cramp M. Dhawan A. (2014). Addressing liver disease in the UK: a blueprint for attaining excellence in health care and reducing premature mortality from lifestyle issues of excess consumption of alcohol, obesity, and viral hepatitis. *Lancet* 384 1953–1997. 10.1016/s0140-6736(14)61838-61839 25433429

[B142] Wu H. Tao J. Li X. Zhang T. Zhao L. Wang Y. (2017). MicroRNA-206 prevents the pathogenesis of hepatocellular carcinoma by modulating expression of met proto-oncogene and cyclin-dependent kinase 6 in mice. *Hepatology* 66 1952–1967. 10.1002/hep.29374 28714063 PMC5696004

[B143] Wu J. R. Hu C. T. You R. I. Pan S. M. Cheng C. C. Lee M. C. (2015). Hydrogen peroxide inducible clone-5 mediates reactive oxygen species signaling for hepatocellular carcinoma progression. *Oncotarget* 6 32526–32544. 10.18632/oncotarget.5322 26416447 PMC4741710

[B144] Xiang Q. F. Zhan M. X. Li Y. Liang H. Hu C. Huang Y. M. (2019). Activation of MET promotes resistance to sorafenib in hepatocellular carcinoma cells via the AKT/ERK1/2-EGR1 pathway. *Artif. Cells Nanomed. Biotechnol.* 47 83–89. 10.1080/21691401.2018.1543195 30663411

[B145] Xie B. Xing R. Chen P. Gou Y. Li S. Xiao J. (2010). Down-regulation of c-Met expression inhibits human HCC cells growth and invasion by RNA interference. *J. Surg. Res.* 162 231–238. 10.1016/j.jss.2009.04.030 19765730

[B146] Xie Q. Liu K. D. Hu M. Y. Zhou K. (2001). SF/HGF-c-Met autocrine and paracrine promote metastasis of hepatocellular carcinoma. *World J. Gastroenterol.* 7 816–820. 10.3748/wjg.v7.i6.816 11854908 PMC4695601

[B147] Xue X. Wang X. Zhao Y. Hu R. Qin L. (2018). Exosomal miR-93 promotes proliferation and invasion in hepatocellular carcinoma by directly inhibiting TIMP2/TP53INP1/CDKN1A. *Biochem. Biophys. Res. Commun.* 502 515–521. 10.1016/j.bbrc.2018.05.208 29859935

[B148] Yamagamim H. Moriyama M. Matsumura H. Aoki H. Shimizu T. Saito T. (2002). Serum concentrations of human hepatocyte growth factor is a useful indicator for predicting the occurrence of hepatocellular carcinomas in C-viral chronic liver diseases. *Cancer* 95 824–834. 10.1002/cncr.10732 12209727

[B149] Yan S. Shan X. Chen K. Liu Y. Yu G. Chen Q. (2018). LINC00052/miR-101-3p axis inhibits cell proliferation and metastasis by targeting SOX9 in hepatocellular carcinoma. *Gene* 679 138–149. 10.1016/j.gene.2018.08.038 30098428

[B150] Yang J. Lu Y. Lin Y. Y. Zheng Z. Y. Fang J. H. He S. (2016). Vascular mimicry formation is promoted by paracrine TGF-beta and SDF1 of cancer-associated fibroblasts and inhibited by miR-101 in hepatocellular carcinoma. *Cancer Lett.* 383 18–27. 10.1016/j.canlet.2016.09.012 27693460

[B151] Yang X. Zhang X. F. Lu X. Jia H. L. Liang L. Dong Q. Z. (2014). MicroRNA-26a suppresses angiogenesis in human hepatocellular carcinoma by targeting hepatocyte growth factor-cMet pathway. *Hepatology* 59 1874–1885. 10.1002/hep.26941 24259426

[B152] Yang Y. M. Lee C. G. Koo J. H. Kim T. H. Lee J. M. An J. (2015). Galpha12 overexpressed in hepatocellular carcinoma reduces microRNA-122 expression via HNF4alpha inactivation, which causes c-Met induction. *Oncotarget* 6 19055–19069. 10.18632/oncotarget.3957 25965999 PMC4662475

[B153] Yau T. C. C. Lencioni R. Sukeepaisarnjaroen W. Chao Y. Yen C. J. Lausoontornsiri W. (2017). A Phase I/II multicenter study of single-agent foretinib as first-line therapy in patients with advanced hepatocellular carcinoma. *Clin. Cancer Res.* 23 2405–2413. 10.1158/1078-0432.Ccr-16-1789 27821605 PMC5420486

[B154] You H. Ding W. Dang H. Jiang Y. Rountree C. B. (2011). c-Met represents a potential therapeutic target for personalized treatment in hepatocellular carcinoma. *Hepatology* 54 879–889. 10.1002/hep.24450 21618573 PMC3181384

[B155] Zhang H. Liao Z. Liu F. Su C. Zhu H. Li Y. (2019). Long noncoding RNA HULC promotes hepatocellular carcinoma progression. *Aging* 11 9111–9127. 10.18632/aging.102378 31645479 PMC6834430

[B156] Zhang J. P. Zeng C. Xu L. Gong J. Fang J. H. Zhuang S. M. (2014). MicroRNA-148a suppresses the epithelial-mesenchymal transition and metastasis of hepatoma cells by targeting Met/Snail signaling. *Oncogene* 33 4069–4076. 10.1038/onc.2013.369 24013226

[B157] Zhang Y. S. Chu J. H. Cui S. X. Song Z. Y. Qu X. J. (2014). Des-gamma-carboxy prothrombin (DCP) as a potential autologous growth factor for the development of hepatocellular carcinoma. *Cell Physiol. Biochem.* 34 903–915. 10.1159/000366308 25200250

[B158] Zhang K. Zhao Z. Yu J. Chen W. Xu Q. Chen L. (2018a). LncRNA FLVCR1-AS1 acts as miR-513c sponge to modulate cancer cell proliferation, migration, and invasion in hepatocellular carcinoma. *J. Cell. Biochem.* 119 6045–6056. 10.1002/jcb.26802 29574975

[B159] Zhang S. Z. Pan F. Y. Xu J. F. Yuan J. Guo S. Y. Dai G. (2005). Knockdown of c-Met by adenovirus-delivered small interfering RNA inhibits hepatocellular carcinoma growth in vitro and in vivo. *Mol. Cancer Ther.* 4 1577–1584. 10.1158/1535-7163.Mct-05-0106 16227408

[B160] Zhang Y. Gao X. Zhu Y. Kadel D. Sun H. Chen J. (2018b). The dual blockade of MET and VEGFR2 signaling demonstrates pronounced inhibition on tumor growth and metastasis of hepatocellular carcinoma. *J. Exp. Clin. Cancer Res.* 37:93. 10.1186/s13046-018-0750-752 29712569 PMC5925844

[B161] Zhang Y. Xia M. Jin K. Wang S. Wei H. Fan C. (2018c). Function of the c-Met receptor tyrosine kinase in carcinogenesis and associated therapeutic opportunities. *Mol. Cancer* 17:45. 10.1186/s12943-018-0796-y 29455668 PMC5817860

[B162] Zhang Y. W. Su Y. Volpert O. V. Vande Woude G. F. (2003). Hepatocyte growth factor/scatter factor mediates angiogenesis through positive VEGF and negative thrombospondin 1 regulation. *Proc. Natl. Acad. Sci. U.S.A.* 100 12718–12723. 10.1073/pnas.2135113100 14555767 PMC240684

[B163] Zhu A. X. Kang Y. K. Yen C. J. Finn R. S. Galle P. R. Llovet J. M. (2019). Ramucirumab after sorafenib in patients with advanced hepatocellular carcinoma and increased alpha-fetoprotein concentrations (REACH-2): a randomised, double-blind, placebo-controlled, phase 3 trial. *Lancet Oncol.* 20 282–296. 10.1016/s1470-2045(18)30937-30939 30665869

[B164] Zhuang P. H. Xu L. Gao L. Lu W. Ruan L. T. Yang J. (2017). Correlations of microvascular blood flow of contrast-enhanced ultrasound and HGF/c-Met signaling pathway with clinicopathological features and prognosis of patients with hepatocellular carcinoma. *Oncol. Targets Ther.* 10 847–857. 10.2147/ott.S113353 28243120 PMC5317332

